# Nonlinear photonics on integrated platforms

**DOI:** 10.1515/nanoph-2024-0149

**Published:** 2024-06-26

**Authors:** Wenpu Geng, Yuxi Fang, Yingning Wang, Changjing Bao, Weiwei Liu, Zhongqi Pan, Yang Yue

**Affiliations:** Institute of Modern Optics, Nankai University, Tianjin 300350, China; Department of Electrical Engineering, University of Southern California, Los Angeles, CA 90089, USA; Department of Electrical & Computer Engineering, University of Louisiana at Lafayette, Lafayette, LA 70504, USA; School of Information and Communications Engineering, 12480 Xi’an Jiaotong University, Xi’an 710049, China

**Keywords:** nonlinear photonics, integrated photonics, frequency combs, supercontinuum generation

## Abstract

Nonlinear photonics has unveiled new avenues for applications in metrology, spectroscopy, and optical communications. Recently, there has been a surge of interest in integrated platforms, attributed to their fundamental benefits, including compatibility with complementary metal-oxide semiconductor (CMOS) processes, reduced power consumption, compactness, and cost-effectiveness. This paper provides a comprehensive review of the key nonlinear effects and material properties utilized in integrated platforms. It discusses the applications and significant achievements in supercontinuum generation, a key nonlinear phenomenon. Additionally, the evolution of chip-based optical frequency combs is reviewed, highlighting recent pivotal works across four main categories. The paper also examines the recent advances in on-chip switching, computing, signal processing, microwave generation, and quantum applications. Finally, it provides perspectives on the development and challenges of nonlinear photonics in integrated platforms, offering insights into future directions for this rapidly evolving field.

## 1 Introduction

Since the first observation of laser-driven nonlinear optics phenomenon [1], the field of nonlinear optics surged forward, driven by practical applications in various industries and disciplines. The incidence of intense beams can alter the optical properties of the medium, resulting in variations in the spectral, spatial, or polarization properties of the light beam, or the generation of new frequency components in unconventional spectral windows [2]. With ongoing in-depth research, numerous nonlinear effects have emerged and the high-quality output spectra are pursued.

**Figure 1:**
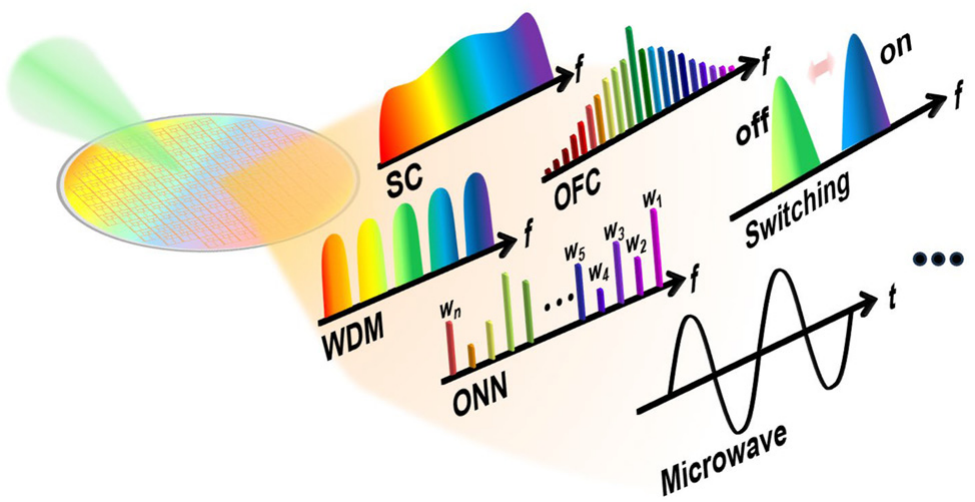
Typical applications of the nonlinear photonics on integrated platforms. SC, supercontinuum; OFC, optical frequency comb; WDM, wavelength division multiplexing; ONN, optical neural networks.

With the emergence of “integrated optics,” a significant turning point in the history of photonics, the research directions of nonlinear optics have been expanded notably, shifting from free space or optical fibers as propagation media toward compact on-chip integration techniques. Integrated photonic devices confine the optical field within areas comparable to the wavelength of light, maintaining strong optical field intensities, high power densities, and longer interaction lengths between light and material. These characteristics enable the triggering and utilization of nonlinear effects at relatively low power levels, significantly reducing the energy threshold needed for nonlinear phenomena. Such efficient nonlinear interactions open new avenues for research and applications, including supercontinuum (SC) generation, optical frequency comb (OFC) generation, and all-optical switching as depicted in Figure 1. Another significant advantage of integrated photonic devices is their small footprint, which reduces manufacturing costs and enables large-scale integration of optical components on a single chip. This advancement significantly paves the development of all-optical computing and communication technologies, allowing for more complex and functional optical systems to be designed and deployed in smaller form factors.

**Figure 2:**
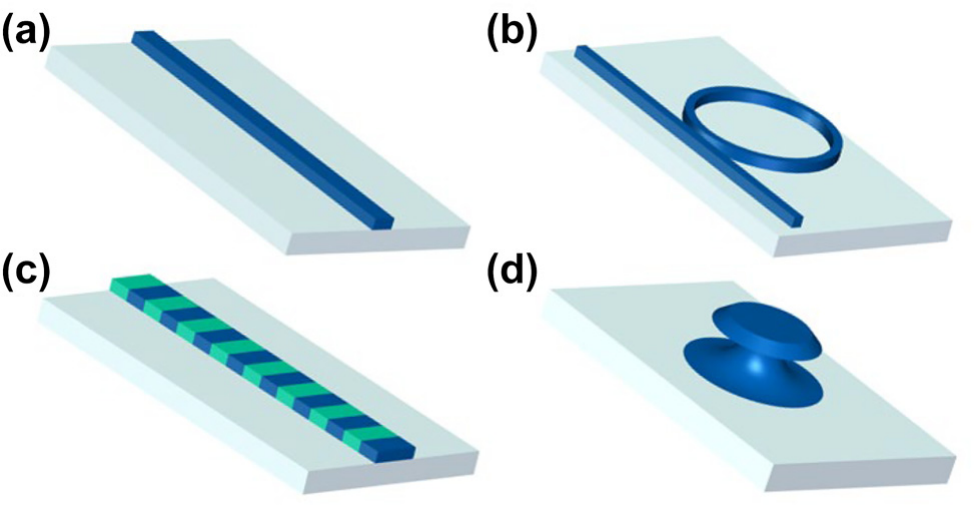
Schematic diagram of several integrated nonlinear photonic structures: (a) waveguide, (b) microring resonator, (c) periodically poled waveguide, and (d) microdisk resonator.

The development of materials and fabrication techniques that support strong nonlinear optical responses and high optical quality factors is of vital importance in the advancement of integrated nonlinear photonics. Numerous novel materials have been reported for nonlinear photonics on integrated platform. Figure 2 provides the classical structures of integrated nonlinear photonics. Optical waveguide, depicted in Figure 2(a), serves as a fundamental element and cornerstone for various integrated photonic devices, including the microring resonator in Figure 2(b), the periodically poled lithium niobate (PPLN) in Figure 2(c), and the microdisk resonator in Figure 2(d). The corresponding principles and applications of these structures will be discussed in the following sections. Furthermore, there are also various structures in integrated photonics, such as subwavelength gratings and photonic crystal cavities. With ongoing advancements in manufacturing techniques and the utilization of new materials, the field of integrated photonics is expected to continue its rapid development, offering more powerful and flexible solutions for future optoelectronic systems.

In this paper, we provide a comprehensive review of recent advancements in nonlinear photonics on integrated platforms and applications envisioned therein. The rest of the paper is organized as follows: In Section 2, a general introduction to nonlinear effects involved in integrated photonics is introduced, including the second-order and third-order nonlinear processes. Since material properties play a crucial role in determining the operating bandwidth, efficiency, and compatibility of integrated photonic devices, we also delve into material properties relevant to integrated nonlinear photonics in this section. This includes discussions on transparency windows, refractive indices, and nonlinear refractive indices. In Section 3, the applications and the representative results of supercontinuum generation (SCG) in integrated platforms are discussed. In Section 4, we present the history of chip-based optical frequency comb and highlights recent representative works. The applications of integrated nonlinear photonics, including all-optical switching, data communication, optical neural network, microwave generation, and more, are discussed in Section 5 with recent progress.

## 2 Nonlinear optical processes and materials

**Figure 3:**
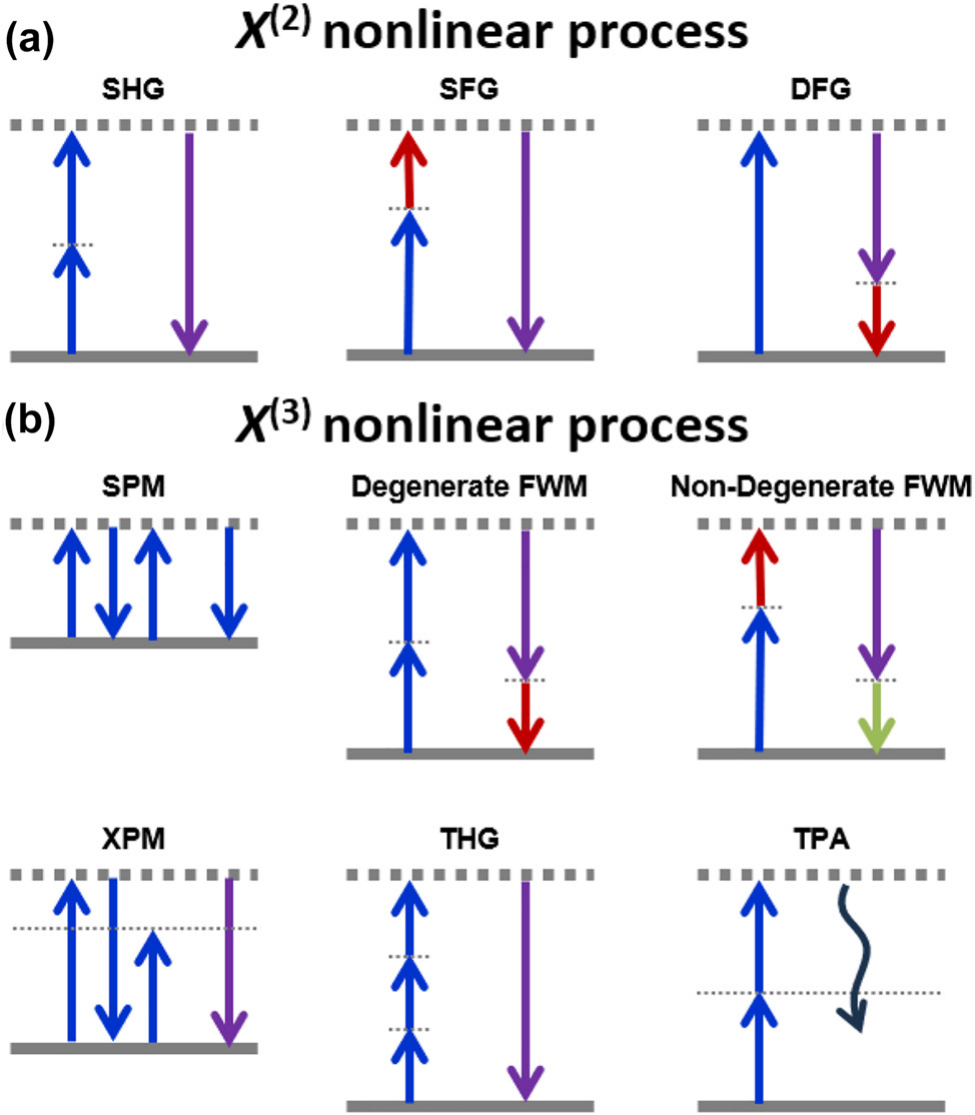
Schematic illustration of (a) second-order nonlinear (*χ* ^(2)^) and (b) third-order (*χ* ^(3)^) nonlinear optical processes in integrated platforms. SHG, second-harmonic generation; SFG, sum frequency generation; DFG, difference frequency generation; SPM, self-phase modulation; FWM, four-wave mixing; XPM, cross-phase modulation; THG, third-harmonic generation; TPA, two-photon absorption.

Nonlinear optics involves the study of the material nonlinear response to intense electromagnetic fields. This nonlinear response stems from the anharmonic motion of bound electrons when subjected to an externally enhanced field. The phenomena of nonlinear optics are typically manifested in the relationship between the material total polarization (*P*) and the electric field (*E*) as follows

(1) $$P=\varepsilon_{0}\left(\chi^{\left(1\right)}\cdot E+\chi^{\left(2\right)}:EE+\chi^{\left(3\right)}\vdots EEE+\cdots \;\right)$$

where *ε*_0_ represents the vacuum permittivity and *χ* ^(*i*)^ denotes the *i*th order susceptibility. The linear first-order susceptibility *χ* ^(1)^ correlates with the refractive index and attenuation or gain within the material. Nonlinear optics delves into the intricate relationship between polarization and the higher-order nonlinear susceptibilities. Figure 3 summarizes the typical nonlinear optical processes.

The second-order susceptibility *χ* ^(2)^ leads to the second-order nonlinearity effects, including second-harmonic generation (SHG) [1], [3], [4], sum-frequency generation (SFG) [5]–[7], and difference frequency generation (DFG) [8]–[10]. The first discovery of the second harmonic marked the beginning of nonlinear optics research. SHG is characterized by the interaction of two waves at the same frequency *ω*_1_ (in practical applications usually only one input beam is required) that produces a wave at 2*ω*_1_. SFG occurs when a signal wave at frequency *ω*_1_ mixes with a pump wave at frequency *ω*_2_, resulting in a harmonic oscillation at *ω*_3_ = *ω*_1_ + *ω*_2_. This process is applied in frequency up-conversion, especially in applications needing the conversion of visible or infrared light to ultraviolet light. In DFG, a difference frequency wave at *ω*_3_ = *ω*_1_ − *ω*_2_ is generated from two waves at frequency *ω*_1_ and *ω*_2_, accompanied by signal amplification. DFG plays a pivotal role in various nonlinear processes, including optical parametric amplification (OPA) and optical parametric oscillator (OPO). Additionally, Pockels effect, as the linear electro-optic effect, can linearly modify the refractive index of a material by applying an electric field. However, numerous media possess inversion symmetry and the corresponding *χ* ^(2)^ is zero. In contrast to the broadly applicable third-order nonlinearity, the range of on-chip materials suitable for harnessing second-order nonlinearity is more finite, including options such as lithium niobate [11], aluminum nitride [12], and AlGaAs [13].

Third-order nonlinearities stems from the third-order susceptibility *χ* ^(3)^, including a vast array of third-order nonlinear effects such as self-phase modulation (SPM) [14]–[18], four-wave mixing (FWM) [19]–[23], cross-phase modulation (XPM) [24]–[26], third-harmonic generation (THG) [27]–[30], and two-photon absorption (TPA) [31].

As input pulses propagate through a highly nonlinear waveguide, the initial temporal and spectral evolutions are predominantly influenced by the SPM effect. This effect originates from dipole excitations triggered by the interaction of three photons. SPM leads to variations in intensity-dependent refractive index, consequently altering the spectral composition of the pulse responsible for this change [32]. SPM is one of the primary effects contributing to supercontinuum generation, which significantly broadens the incident short pulse in the frequency domain.

FWM facilitates the transfer of energy from two pump waves (*ω*_1_ and *ω*_2_) to two distinct frequencies (*ω*_3_ and *ω*_4_). Additionally, when a weak signal at *ω*_3_ is introduced alongside the pump waves, it gets amplified while simultaneously generating a new wave at *ω*_4_. Nondegenerate FWM, as the general case, involves the mixing of three different frequencies (*ω*_1_ ≠ *ω*_2_ ≠ *ω*_3_). In cases where *ω*_1_ equals *ω*_2_, known as degenerate FWM, the process can be activated by a single pump beam. The energy is transferred from a strong pump wave to the upshifted and downshifted frequency components. FWM has found extensive applications in parametric amplification [33], wavelength conversion [34], and quantum correlation [35].

The occurrence of XPM stems from the phase modulation of signal *ω*_2_ induced by the optical intensity of the copropagating signal *ω*_1_. At equal intensities, XPM exhibits twice the effectiveness of SPM. The THG process involves generating a wave at frequency *ω*_4_ = *ω*_1_ + *ω*_2_ + *ω*_3_ by mixing three waves of the same frequency (*ω*_1_ = *ω*_2_ = *ω*_3_). In general, fulfilling the phase-matching condition for this process poses a challenge.

When subjected to a pump pulse of high intensity, nonlinear absorption may result from two-photon absorption (TPA), leading to a reduction in spectral intensity. TPA process will induce a significant generation of free carriers (electrons and holes). These free carriers not only absorb light but also influence the refractive index. The related phenomena are known as free carrier absorption (FCA) and free carrier dispersion (FCD), respectively.

**Figure 4:**
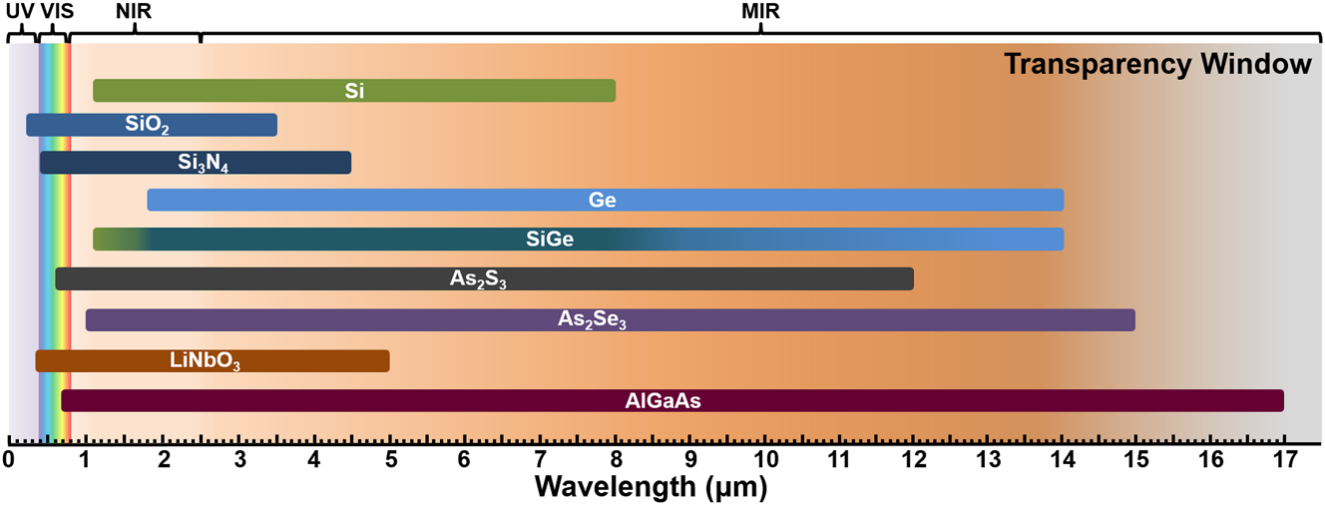
Transparency window of different integrated platforms. UV, ultraviolet; VIS, visible; NIR, near infrared; MIR, mid infrared.

Given that nonlinear effects arise from the response of materials to intense electromagnetic fields, knowledge of the properties of various nonlinear materials is pivotal for on-chip nonlinear investigations. Table 1 provides the summary of the common platforms for integrated nonlinear photonics, including silicon (Si), silica (SiO_2_), silicon nitride (Si_3_N_4_), germanium (Ge), silicon germanium (SiGe), arsenic trisulfide (As_2_S_3_), arsenic triselenide (As_2_Se_3_), lithium niobate (LiNbO_3_), and aluminum gallium arsenide (AlGaAs). Here, we mainly focus on the band gap energy, transparency window as depicted in Figure 4, refractive index *n* reflecting the dispersion property, and the nonlinear refractive index *n*_2_ representing nonlinear property. For a more detailed review of nonlinear materials, please refer to [36]. The integrated photonics potentially brings new opportunities in tremendous range of scientific discovery and novel applications, such as fluorescence imaging, medicine, and astronomy, leveraging the ultraviolet (UV) and visible (VIS) spectroscopy; telecommunication, information processing, and spectroscopy in the near infrared (NIR) spectroscopy; and the spectroscopy of gases and biomolecules in the mid-infrared (MIR) region.

**Table 1:** The basic properties of platforms for integrated nonlinear photonics. Values of *n* and *n*_2_ are given at 1,550 nm unless stated otherwise.

Material	Bandgap energy (eV)	Transparency window (μm)	*n*	*n*_2_ (10^−17^ m^2^/W)	*χ* ^(2)^
Si	1.12 [37]	1.1 [38] −8 [39]	3.48 [40]	0.56 [38], [41]	
SiO_2_	8.9 [42]	0.2 [43] −3.5 [44]	1.44 [45]	0.0026 [38]	
Si_3_N_4_	5.3 [46]	0.4–4.5 [47], [48]	1.98 [38], [49]	0.024 [50]	
Ge	0.7 [39]	1.8 [51] −14 [39]	4 (at 4 μm) [52]	2.7 (at 4 μm) [38], [41]	
SiGe (Si_*x*_Ge_1−*x*_)	0.82–1.11 [37]	1.1–14 [39]	3.6 (*x* = 0.6) (at 4 μm) [41]	∼0.4 (at 4 μm) [41], [53]	
As_2_S_3_	2.4 [54]	0.6 [43] −12 [55]	2.44 [56]	0.38 [43], [54]	
As_2_Se_3_	1.77 [37]	1 [43] −15 [55]	2.73 [38]	3 (at 1,064 nm) [57]	
LiNbO_3_	4.9 [58]	0.35–5 [59]	2.21(o) 2.14(e)	0.018	√
AlGaAs (Al_*x*_Ga_1−*x*_As)	1.6–1.79 [23]	0.7–17 [43]	3.3 [43]	2 [60]	√

As the most widely used and developed integrated platform, silicon offers a transparency window from 1.1 to 8 μm [38], [39]. Under high optical power pump, Si in the range of 1.1–2.2 μm suffers significant nonlinear absorption due to TPA [31]. Benefiting from the relatively high refractive index of Si (about 3.48) [40], light propagated in it is confined well. The bandgap of Si is 1.12 eV [37] and *n*_2_ is 0.56 × 10^−17^ m^2^/W at 1,550 nm [38], [41]. With the bandgap of 8.9 eV [42], the low-loss transparency window of SiO_2_ is from 0.2 μm [43] to 3.5 μm [44]. SiO_2_ has relatively low material refractive index 1.44 [45] and *n*_2_ 0.0026 × 10^−17^ m^2^/W at 1,550 nm [38], which is the common cladding material. With the bandgap energy of 5.3 eV and a negligible TPA coefficient [46], [47], Si_3_N_4_ enabling an extended transparency window into the short-wavelength region, with coverage from 0.4 to 4.5 μm [47], [48]. Featuring a larger material refractive index than that of SiO_2_ [49], Si_3_N_4_ also exhibits nonlinear refractive index that is one order of magnitude greater [50]. Ge, also group IV element, has an expansive transparency window extending up to 14 μm [39], [51] and material refractive index around 4 [52]. Typically, *n*_2_ of Ge is 2.7 × 10^−17 ^m^2^/W at 4,000 nm [38], [41], which is significantly higher than that of other materials. For the alloy material SiGe, the extent of transparency window is contingent on its material composition, as shown in Figure 4 through the gradient color, allowing for extensions up to 14 μm under low silicon concentrations. The bandgap energy, *n* and *n*_2_ of SiGe, is also determined by the material composition [41]. Chalcogenide glasses, containing As_2_S_3_ and As_2_Se_3_, possess notable transparency ranges, from 0.6 [43] to 12 μm [55] and 1 [43] to 15 μm [55], respectively. Their material refractive indices are between silicon and silicon nitride [38], [56]. The *n*_2_ of As_2_Se is on the same order of magnitude as that of Ge [57]. Lithium niobate, known for its robust *χ* ^(2)^ and moderate *χ* ^(3)^ nonlinearity, has a transparency window spanning from 0.35 to 5 μm [59]. The refractive index of LiNbO_3_ is about 2.2 at 1,550 nm and the nonlinear refractive index is one order of magnitude larger than that of SiO_2_ [59]. Particularly, the phase match and dispersion in structure of PPLN can be engineered independently through periodic poling. Without the centrosymmetric crystal structure, Al_*x*_Ga_1−*x*_As also exhibits strong second-order nonlinearity. In recent years, with the development of fabrication processes, AlGaAs, which has a high refractive index and *n*_2_ [43], [60], has made significant progress in practical applications [61]. In addition, Si and Si_3_N_4_ can also exhibit *χ* ^(2)^ nonlinearity under stress or electric field [43], [62].

Polymer, as another large group of materials [63], has been extensively investigated before 2000. Organic electro-optic (EO) materials consist of dipolar chromophores with asymmetric intramolecular charge distribution. Under the undisturbed state, the dipolar chromophores exhibit random orientations with an almost negligible electro-optic coefficient. After an electric poling process, the dipolar chromophores align along the direction of the poling electric field where a strong Pockels effect occurs [64]. The ultrafast EO response and large EO coefficient (up to hundreds compared to 30.9 pm/V of LiNbO_3_) make polymer excellent materials for high perform EO modulators [65], [66]. In this paper, we mainly focus on the inorganic materials.

The materials discussed exhibit significant differences in transparency windows, refractive indices, and nonlinear refractive indices. For example, using SiO_2_ as claddings or substrates could lead to high energy consumption when the wavelength exceeds 3.5 μm. In addition to considering the transparency window, the index contrast between the waveguiding core and the substrate influences the compactness and nonlinear coefficients. Moving beyond traditional materials like silicon dioxide for the substrate and air for the cladding, some studies employ suspended structures to enhance the refractive index contrast further. The summary of cross sections for various types of integrated waveguides is available in Reference [67]. Therefore, selecting an appropriate material combination for a specific application is essential. Additionally, careful consideration of material compatibility during device design and fabrication is crucial. In addition to the commonly used materials mentioned above, some novel materials have also been proposed for specific applications, such as aluminum nitride (AlN), tantalum pentoxide (Ta_2_O_5_), silicon carbide (SiC), and gallium phosphide (GaP). In subsequent sections, we will introduce these materials in their application scenarios.

## 3 Supercontinuum generation

Integrated nonlinearity enhances performance and enables miniaturization across diverse optics-based applications, particularly through supercontinuum generation and optical frequency combs generation that provide coherent, ultra-broadband light sources. Supercontinuum generation (SCG) is a nonlinear phenomenon occurring when an intense optical pulse propagates through a nonlinear medium. The interplay of the dispersive properties and nonlinear effects in the medium contributes to significant spectral broadening. This section focuses on the application and representative works of SCG in integrated photonic waveguides.

**Figure 5:**
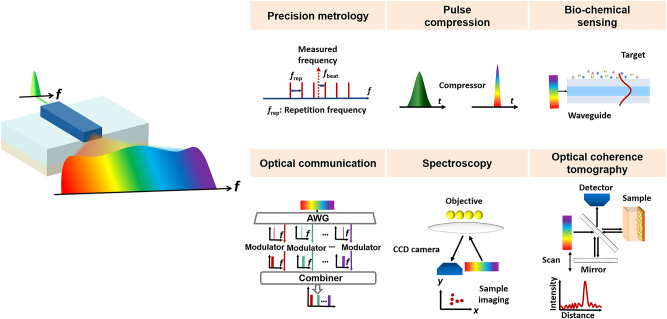
Typical applications based on SCG.

Since its initial discovery in the 1970s, SCG has garnered considerable interest for various potential applications as depicted in Figure 5. Frequency metrology enables the measurement of unknown frequencies through a comb by determining the beat note frequency between a known optical reference and the measured frequency. SCG can serve as an effective approach to extend frequency comb to octave spanning, refining the accuracy of frequency metrology [68]. SC can replace multiple lasers in wavelength division multiplexing (WDM) systems with a single source for various wavelength channels [69]–[71]. The capacity of electrical time-division multiplexing systems [72] can be enhanced through pulse compression. This technique employs SCG to expand the spectrum of input pulses, followed by their subsequent recompression in the time domain [73]–[75]. Optical coherence tomography (OCT), which captures cross-sectional images of micrometer-scale objects, represents a key application of SCG [76]–[79]. The supercontinuum light source is focused on a small spot, and the OCT system detects the depth information of the sample by scanning the reference arm of the interferometer. Utilizing the source with broadband output spectrum, the resolution of OCT can be significantly improved. The OCT system using a millimeter-scale integrated SC source has been reported [80]. Supercontinuum light paves the way for future sensing applications in biological [81]–[83], chemical [84], [85], and environmental [86], [87]. The expansive bandwidth of SC-enhanced mid-infrared (MIR) sources aids in broadening the spectrum of detectable molecules for spectroscopic analysis. The broadband spectrum finds applications in molecular spectroscopy, including single nanoparticles identification [88], molecular fingerprinting [89], [90], absorption spectroscopy [91]–[93], etc.

In this section, we explore representative results in SCG utilizing various materials. Table 2 shows the summary of the corresponding key factors that impact the spectral broadening, including the length, nonlinear coefficient *γ* and loss *α* of the waveguide, pump wavelength *λ*_p_, and width of the input pulse.

**Table 2:** Parameters in SCG process of waveguides with various materials.

Material	Length (mm)	*λ*_p_ (μm)	Peak power (kW)	Pulse width (fs)	*γ* (m^−1^W^−1^)	*α* (dB/cm)	Wavelength range (μm)
SOI [94]	12	4	96	300	0.41 [67]	5	2–5 @−30 dB level
SOI [95]	10	2.29	0.225	70	38	<0.2	1.54–3.2 @−30 dB level
SOS [96]	16	3.7	1.82	320	8.86^b^	1	1.9–5.5 @−30 dB level
SiO_2_ [97]	15	1.064	25.56	90	0.038 [67]	N.A.	0.5–1.5 @−40 dB level
Si_3_N_4_ [48]	5	1.55	>11	<90	0.82 [67]	0.2 [98]	0.56–3.6 @−40 dB level
Ge [99]	22	4.6	3.3	200	1.59	1.25	3.53 to 5.83 @−30 dB level
SiGe [100]	23 (tapered)	3.9	4.5	200	0.37–1	0.12–1	2.4–5.5 (7.8 from simulation) @−30 dB level
As_2_S_3_ [101]	5	1.97	17^b^	412	4.1	1	0.6–2.8 @−40 dB level
As_2_Se_3_ [102]^a^	6	2.8	6.4	497	28.17	0.0065	1–10.9 @−30 dB level
LiNbO_3_ [103]	10	1.56	4	200	0.4	0.16	0.7–2.2 @−40 dB level
LiNbO_3_ [104]	6.6	1.55	0.69^b^	∼130 fs	0.67^b^	1.1	0.33–2.4 @−40 dB level
AlGaAs [105]	3	1.555	0.036^b^	100	630	2	1.055–2.155 @−40 dB level

^a^Represents the simulation work; ^b^Represents the parameters we calculate indirectly from known parameters.

**Figure 6:**
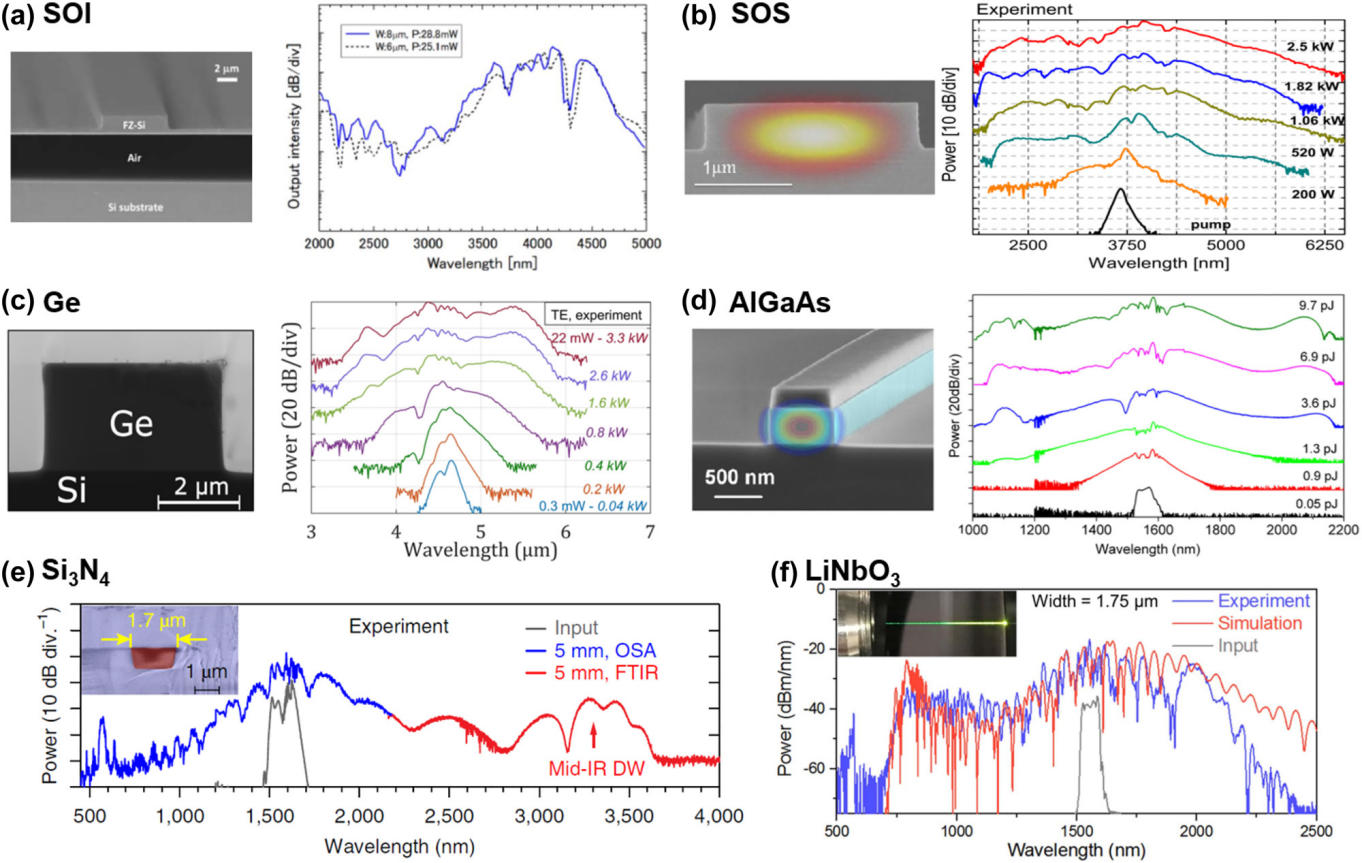
Scanning electron micrograph (SEM) and output spectra of SCG based on different integrated platforms: (a) SOI platform with varying waveguide width *W* and pump power *P* [94]. Adapted with permission from [94] © Optica Publishing Group. (b) SOS platform with varying input peak power [96]. Adapted with permission from [96] © Optica Publishing Group. (c) Ge platform with different peak power [99]. Adapted from [99] under a CC-BY license. (d) AlGaAs with different pump pulse energies [105]. Adapted with permission from [105] © Optica Publishing Group. (e) Si_3_N_4_ platform [48]. From [48]. Adapted with permission from Springer Nature. (f) LiNbO_3_ platform [103]. Adapted with permission from [103] © Optica Publishing Group.

Silicon is widely applied in integrated photonics due to the same manufacturing process flow as complementary metal oxide semiconductor (CMOS) electronics [98]. In addition, the large material refractive index enhances the mode field confinement by the waveguide, enabling flexible dispersion management and compact device size. Therefore, supercontinuum generation in silicon is highly preferred [106], [107]. Silicon-on-insulator (SOI) is the common structure [95], [108], [109]. Figure 6(a) exhibits a supercontinuum generation spans from 2 to 5 μm by pumping a fully suspended SOI waveguide in the anomalous dispersion regime [94]. The figure illustrates the spectral profiles measured for two waveguide widths (*W*), 6 μm and 8 μm, with input powers of 25.1 mW and 28.8 mW, respectively. The resulting spectrum reflects the characteristics of spectrum broadening caused by soliton fission. This phenomenon, widely investigated for its capacity to generate dispersive waves (DW), plays a crucial role in expanding the spectrum toward the short or long wavelength. Nonetheless, this process also introduces spectral fluctuations, which can impact the flatness of the spectrum. With a transparency window beyond 5 μm of the sapphire substrate, silicon-on-sapphire (SOS) is a promising platform for SCG in the mid-infrared range [96], [110]. Singh et al. [96] demonstrated an octave-spanning supercontinuum in SOS nanowires, covering a continuous spectral range from 2 to 6 μm as depicted in Figure 6(b). Furthermore, theoretical findings indicate that a supercontinuum spectrum extending to 8 μm is achievable, offering a potential approach to maximize the use of Si transparency window.

With the broad transparency window from 1.8 to 14 μm, Ge-based platforms are ideal candidates for mid-infrared photonics. The large Kerr coefficient contributes to strong third-order nonlinearity over this range [41]. The generation of SC in pure germanium waveguides was earlier predicted by numerical simulations [111], [112]. Torre et al. experimentally demonstrated SCG spanning nearly an octave from 3.53 μm to 5.83 μm in a low-loss Ge-on-Si waveguide as illustrated in Figure 6(c) [99]. Numerical analyses indicate that free carrier absorption significantly restricts additional expansion of SC into the mid-infrared spectrum.

With an *n*_2_ on the order of 10^−17^ m^2^/W [13], AlGaAs also possesses the second-order nonlinear coefficient [113]. Chiles et al. demonstrated SHG and supercontinuum generation from femtosecond laser sources in suspended AlGaAs waveguides [114]. Kuyken et al. demonstrated octave-spanning SCG on the AlGaAs-on-insulator platform by the emission of two dispersive waves at around 1,100 and 2,100 nm [105] as depicted in Figure 6(d). As the pump pulse energy increases, the SC spectrum broadening was limited by three-photon absorption. The coherence of the output spectrum was also confirmed by interferometric measurements near the pump wavelength.

Si_3_N_4_, with the relatively low loss and a negligible TPA coefficient, is a promising nonlinear alternative to Si [67]. Diverse structures of Si_3_N_4_ waveguides have already been reported for SCG. With the adjustable dispersion and low loss, various nonlinear phenomena can be observed in Si_3_N_4_ waveguides [115]–[118] and the output spectrum can cover the visible wavelength [119], [120]. Figure 6(e) shows beyond two octaves SCG from 0.56 μm to 3.6 μm in Si_3_N_4_ waveguide reported by Guo et al. [48]. Pumped under the anomalous dispersion, the solitons generated during pulse propagation split and emitted red-shifted and blue-shifted DW in the normal dispersion regimes. With relatively large *χ* ^(2)^ nonlinearity, lithium niobate exhibits potential in SCG [103], [121], [122]. Lu et al. [103] reported octave-spanning SCG in lithium niobate waveguides as depicted in Figure 6(f). Tunable dispersive waves and SHG both could be observed by varying waveguide parameters. In addition to the work mentioned above, other materials have also been used in SCG and exhibit outstanding properties, such as SiO_2_ [97], SiGe [53], [100], [123], As_2_S_3_ [101], [124], As_2_Se_3_ [102], InGaP [125], GeAsSe [126], and GeSbS [127].

## 4 Optical frequency comb

**Figure 7:**
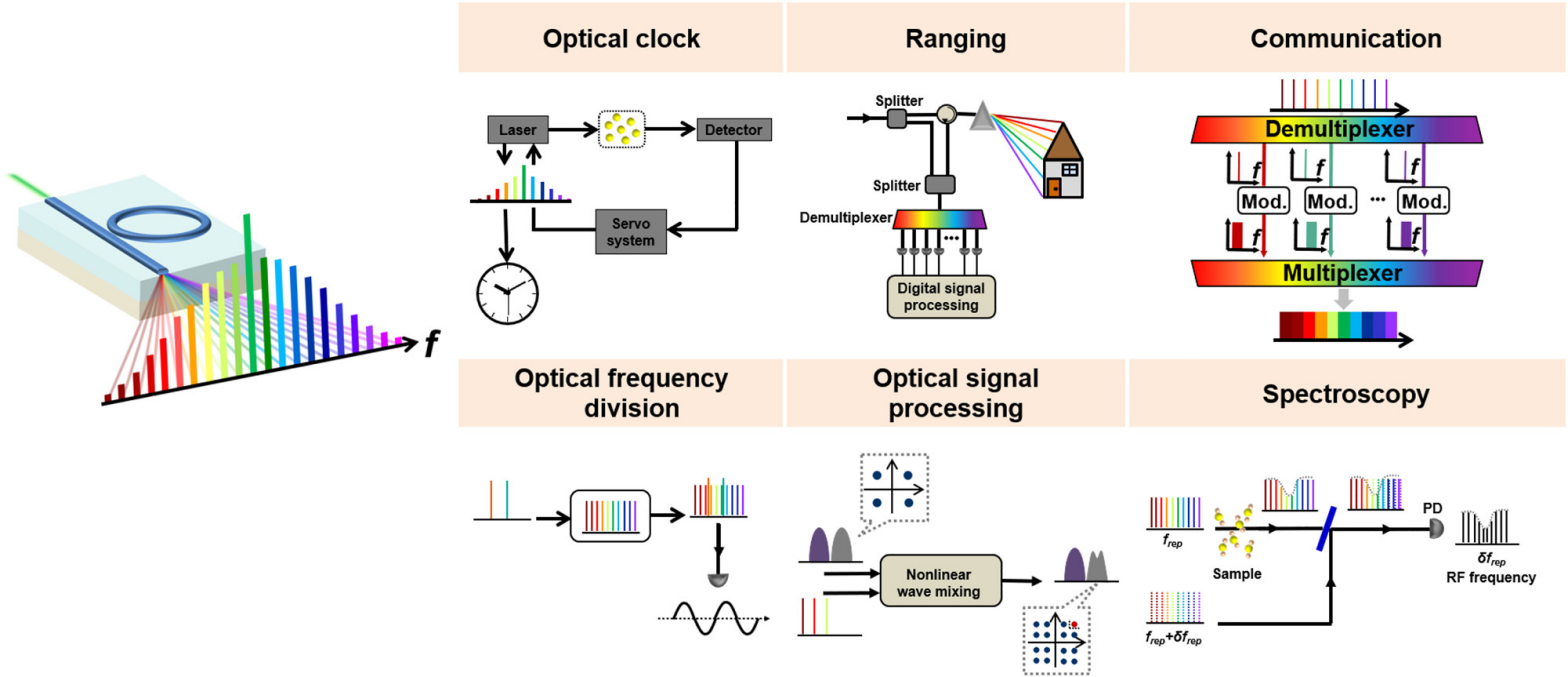
Typical applications based on OFC.

An optical frequency comb is made up of a series of frequency components that are evenly spaced and maintain a stable phase relationship. In 2005, John L. Hall and Theodor W. Hansch, as pioneers of OFC, were awarded the Nobel Prize in Physics for their contributions to the development of laser precision spectroscopy. OFCs serve as precise tools for measuring optical frequencies, effectively bridging the gap between radio, microwave frequencies, and optical frequencies, significantly advancing the field of time-frequency metrology [128]–[130]. The intensity and phase of each frequency line can be adjusted, allowing for the generation of arbitrary optical waveforms in the time domain through Fourier transformation. OFCs can also replace multiple independent lasers, significantly reducing the size, complexity, and energy consumption of communication systems. OFCs have found wide applications across various disciplines such as optical clocks [131]–[133], optical frequency synthesizers [134]–[136], optical communications [137], [138], and spectroscopy [92], [139]–[141], as shown in Figure 7. OFCs can create precise all-optical clocks and achieve optical frequency division by linking the microwave and optical domains. Currently, wavelength division multiplexing, requiring numerous discrete wavelength sources, is the key technology in telecommunications. OFCs, with their equally spaced, phase-locked frequency lines, offer an efficient approach for WDM-based communications. Dual-comb spectroscopy is one of the most common applications of OFCs, where the time-domain interference between two OFCs with slightly different line spacings allows for the rapid and precise conversion of a sample’s absorption spectrum to the radiofrequency domain for detection.

With the advancement of integrated photonics and manufacturing technology, the generation and application of OFCs have moved from bulky laboratory equipment to compact, low-power integrated photonic platforms. In addition to traditional applications, emerging interdisciplinary fields such as optical neural networks [142]–[145], optical signal processing [146], [147], laser-based light detection and ranging (LIDAR) [148], and quantum optics [149]–[151] are on the rise. Microcombs, serving as massively parallel sources on chip, will be discussed in more detail in the following sections.

Technologies enabling the realization of on-chip optical frequency combs cover laser physics, nonlinear optics, electro-optics, among others. The diversity in generation methods underscores the versatility of OFCs, exhibiting their adaptability across a wide range of applications and platforms. Categorized based on the generation mechanisms and platforms, we divide OFCs into four primary types: microresonator based, SC based, electro-optic (EO) based, and mode-locked lasers (MLL) based. This section delves into the historical development and recent advancements of integrated OFCs. Our primary focus lies on microresonator-based OFCs, which leverage optical nonlinear effect to produce comb lines, showing promising potential for practical applications. The remaining three OFC varieties will be briefly discussed, highlighting their respective contributions and advancements in the field.

### 4.1 Microresonator-based OFC

**Figure 8:**
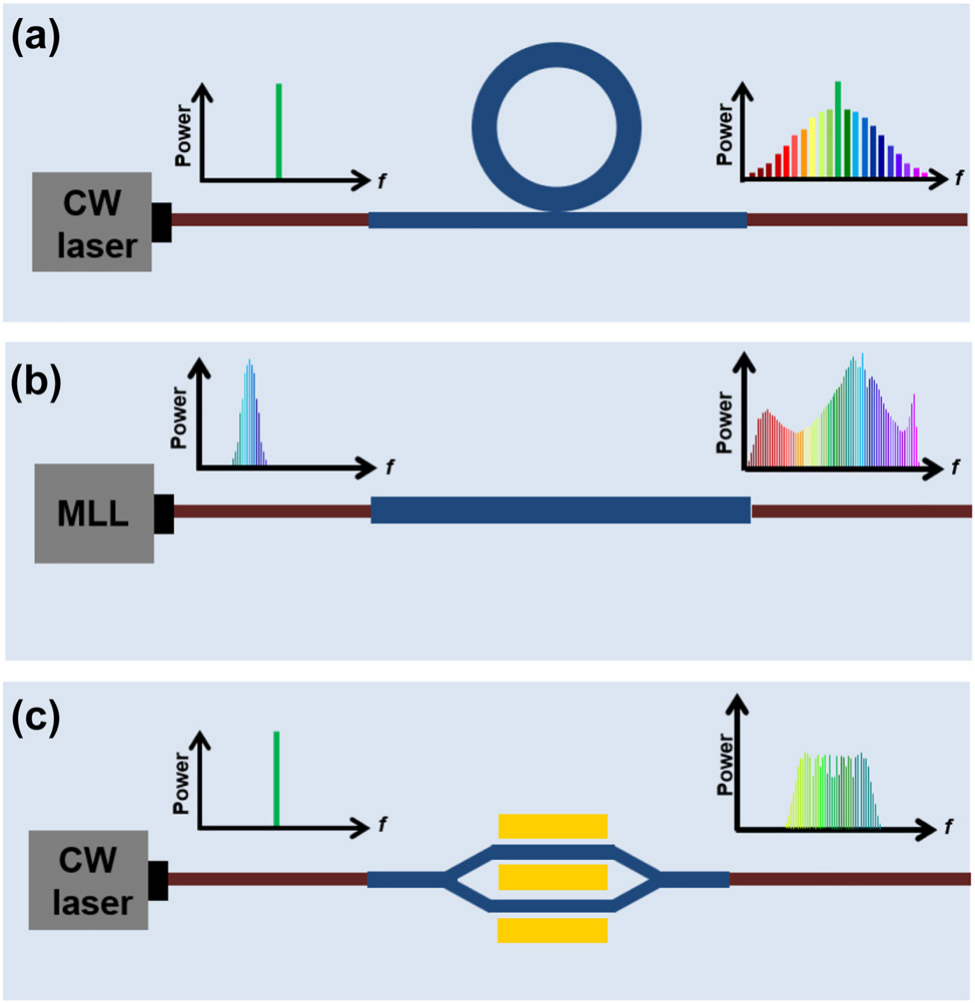
Integrated OFC generation technologies. Schematic configurations of devices for (a) microresonator-based OFC, (b) SC-based OFC, and (c) EO-based OFC.

**Figure 9:**
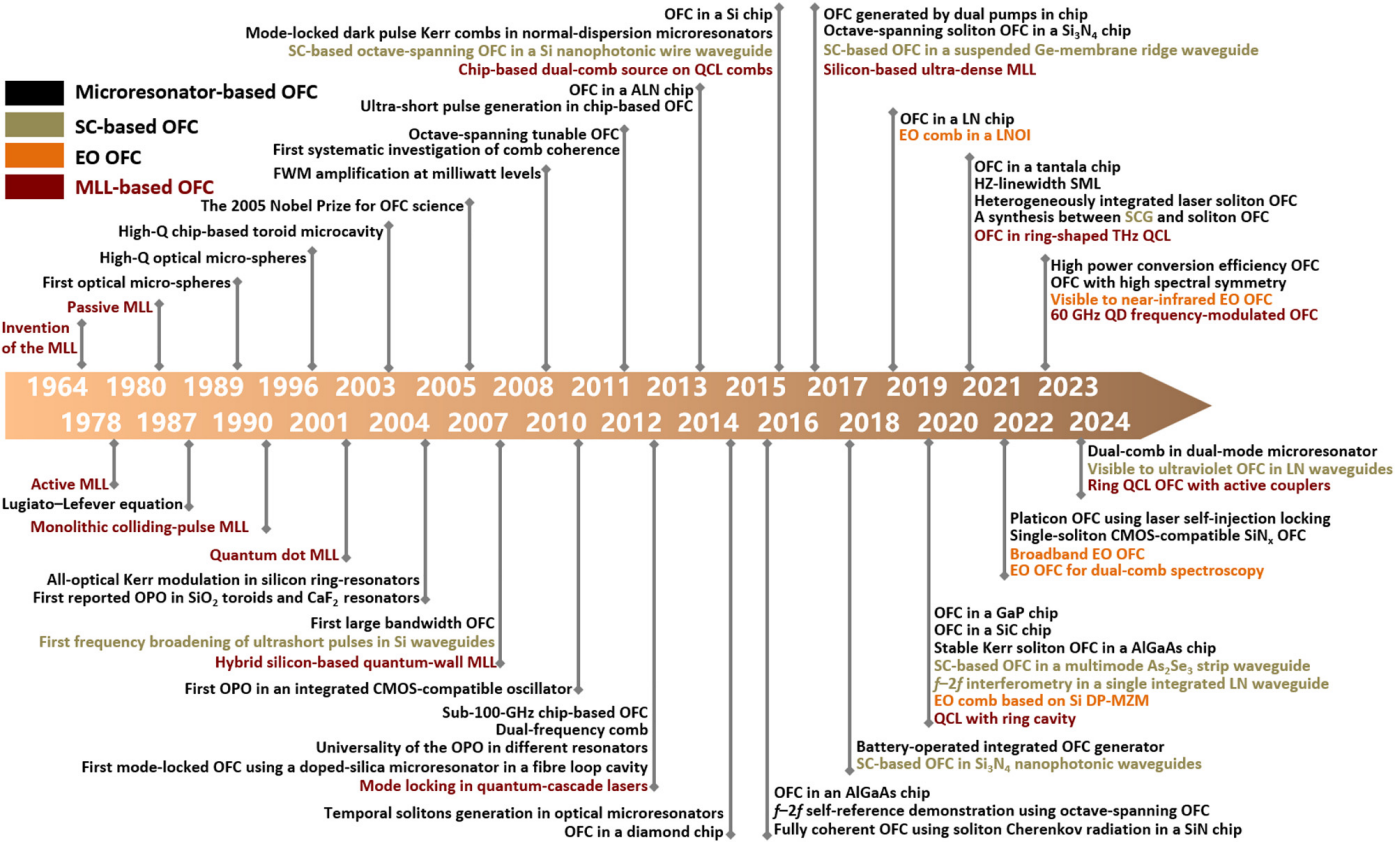
History of OFC based on microresonator, SC, EO effect, and MLL.

Over the past several decades, the exploration of optical frequency combs leveraging microresonators has intensified, driven by their promise for generating effective and compact sources emitting multiple wavelengths. The generation of optical frequency comb in the microresonator is based on the four-wave mixing effect, which is related to the optical Kerr effect, so it is also called Kerr comb. When appropriate dispersion conditions are satisfied, efficient FWM effects can occur between longitudinal modes of microresonators with high quality factors (*Q*). By employing a single-frequency continuous-wave laser to pump the microresonator, crossing a certain power threshold initiates the generation of primary sidebands through degenerate FWM. Subsequent cascading FWM broadens the spectrum, culminating in the creation of an optical frequency comb with equidistant spectral lines as depicts in Figure 8(a) [43]. The repetition rate is governed by the free spectral range (FSR) of the microresonator. Emerging in the late 1980s, the domain of microresonator-based optical frequency combs matured in the early 2000s, propelled by the adoption of whispering-gallery modes across various technological approaches and the integration of chip-based microring resonators for telecommunications. The evolution and advancements in microresonator-based OFC technology are illustrated in Figure 9, marked in black.

In 1989, Braginsky and colleagues reported the initial optical micro-sphere possessing a quality factor exceeding 10^8^ [152], marking a pivotal moment in microresonator research. In 1996, Gorodetsky et al. demonstrated that smoothing the surface can reduce scattering losses, and the quality factor of this kind microresonator reaches 10^10^ [153]. Following this development, various technology and material platforms for microresonators have emerged. As the field of integrated photonics advances, Armani et al. in 2003 introduced the first ultra-high-Q toroid microcavity on the chip [154]. Due to the compact size and high nonlinear coefficients, the light field in microresonators is significantly enhanced and optical nonlinear phenomena can be excited at considerably low power levels [155]. In 2004, Almeida et al. demonstrated all-optical Kerr control in a Si microring resonator [156]. That year also saw the first reports of optical parametric amplification in silica microtoroid and calcium fluoride (CaF_2_) resonators [157], [158], setting the groundwork for future research into microcombs. In 2007, Del’Haye et al. reported OFC with bandwidth up to 500 nm, which sparking interest in utilizing microresonators for chip-based applications [159]. FWM-based frequency conversion was demonstrated in 2008 within milliwatt pumped integrated microring resonators [160], [161]. Optical parametric oscillators based on CMOS-compatible platforms were achieved in 2010 [162], [163], laying the foundation for achieving large-scale integrated on-chip interconnection.

On the established base of theory and technology, later studies have concentrated on improving the performance of frequency combs, specifically in terms of bandwidth, line spacing, and coherence. In 2011, octave-spanning frequency combs in microresonators with different material were first demonstrated [164], [165], which is highly desired to achieve self-referencing with an *f* − 2*f* interferometer in frequencies measurement. The line-by-line pulse shaping and coherence on comb is investigated by Ferdous et al. [166]. In 2012, the first OFC based on integrated platform with repetition rates down to 20 GHz is demonstrated by Johnson et al. [167]. Peccianti et al. reported the first mode-locked Kerr OFC based on a microresonator [168]. The same team also proposed a dual-mode locked laser, exhibiting stable operation of two slightly shifted spectral optical comb replicas [169]. Herr et al. revealed the universal formation dynamics and noise of Kerr-frequency combs in different experimental systems [170]. In 2013, sub-200-fs pulses were generated from a chip-based OFC source, offering a potential for ultrashort laser pulse generation [171]. Observations of temporal dissipative solitons within nonlinear microresonators were made in 2014 [172], and by 2015, Xue and collaborators reported mode-locked dark pulse Kerr combs in silicon nitride microrings under normal dispersion [173]. In 2016, by using soliton Cherenkov radiation, a fully coherent OFC covering 2/3 octave in ring-shaped microresonators was demonstrated [174]. Del’Haye et al. investigated *f* − 2*f* self-referencing of a coherent octave-spanning OFC from microresonator, confirming the application of comb as phase-coherent microwave-to-optical links [175]. In the next year, Bao et al. experimentally generated highly coherent primary combs with multiple sublines by pumping at two wavelengths [176]. Li et al. presented an experimental demonstration of a phase-coherent, octave-spanning soliton optical frequency comb in silicon nitride microring [177].

Stern et al. reported a battery-powered, integrated frequency comb generator in 2018 [178], leveraging developments in heterogeneous fabrication and precision component placement techniques. Following this, in 2021, Jin et al. introduced a microcomb featuring a Hertz-scale linewidth, by self-injection-locking a commercial distributed feedback (DFB) laser to a Si_3_N_4_ microresonator [179]. Anderson et al. demonstrated resonant SCG to bridge the efficiency gap between conventional SCG and soliton microcombs [180]. In the same year, the first demonstration of heterogeneously integrated laser soliton microcombs is reported by Xiang et al. [181]. In 2022, Lihachev et al. demonstrated fully integrated platicon microcomb generation in a DFB pumped integrated Si_3_N_4_ microresonator using laser self-injection locking, which operates at a microwave K-band repetition rate [182]. The first comb with coherent single soliton states on CMOS-compatible low-temperature SiN_*x*_ platforms was reported by Xie et al. [183]. In 2023, to improve the power conversion efficiency, Helgason et al. used two linearly coupled anomalous-dispersion microresonators to induce a controllable frequency shift to a selected cavity resonance. This design allowed the pump to be coupled efficiently into the cavity, resulting in DKS microcombs with a conversion efficiency >50 % [184]. Li et al. reported the frequency comb with high spectral symmetry by introducing two defect modes in microring resonators with inner and outer sidewall gratings [185]. In 2024, to mitigate the instability caused by photo-thermal effect, Miao et al. proposed a dual-comb generation method by pumping two adjacent modes in a microresonator with a single continuous wave (CW) laser [186].

As depicted in Figure 9 and previously mentioned, numerous integrated on-chip platforms capable of generating frequency combs have been demonstrated. In the following sections, we will classify our discussion based on materials to exhibit significant achievements in producing optical frequency combs through microresonators in recent years. This discussion aims to underscore the impact of diverse materials on the advancements within the microresonator-based OFC field. Table 3 summarizes the corresponding structure and *Q* of the microresonator, the pump power coupled into the waveguide, the repetition rate, and wavelength range of the frequency comb. With air as cladding that introduce high refractive index difference, disk and wedge-shaped microresonators exhibit relatively high *Q* factors, while the floating structure also features poor stability and difficulty in coupling [187]. Fabricated by the standard planar process, ring-shaped microresonators are easier to be realized and coupled to the waveguide. However, the cladding materials lead to a lower *Q* factor. Soliton combs with high coherence and low noise are highly favored for practical applications, and the work we reference under soliton states is marked.

**Table 3:** Parameters in microresonator-based OFC generation with various materials.

Material	Structure	Diameter (μm)	Soliton	*Q* factor	Pump power (mW)	Repetition rate (GHz)	Wavelength range (μm)
SiO_2_ [188]	Wedge	3 × 10^3^	√	∼4 × 10^8^	∼180^a^	21.92	1.51–1.6
Si_3_N_4_ [174]	Ring	238	√	∼6 × 10^5a^	2 × 10^3^	189	1.33–2
Si [189]	Ring	200		5.9 × 10^5^	150	127	2.1–3.5
4H-SiC [190]	Ring	100		2.7 × 10^5^	85	∼370^a^	1.475–1.675
4H-SiC [191]	Disk	200		∼6.75 × 10^6^	13	∼260^a^	1.3–1.7
Al_0.2_Ga_0.8_As [192]	Ring	24		1.5 × 10^6^	0.3	1,000	1.45–1.7
LiNbO_3_ [193]	Ring	120	√	∼5 × 10^5a^	240	335	1.2–2.1^a^
Diamond [194]	Ring	40		1 × 10^6^	80	∼925	1.516–1.681^a^
Ta_2_O_5_ [195]	Ring	225	√	∼1 × 10^6a^	33	200	1.3–1.8^a^
GaP [196]	Ring	100		2 × 10^5^	1 × 10^3^	250	1.5–1.62
AlN [197]	Ring	200	√	2.5 × 10^6^	∼1 × 10^3^	∼220	∼1–2.4^a^

^a^Represents the parameters we calculate indirectly from known parameters.

**Figure 10:**
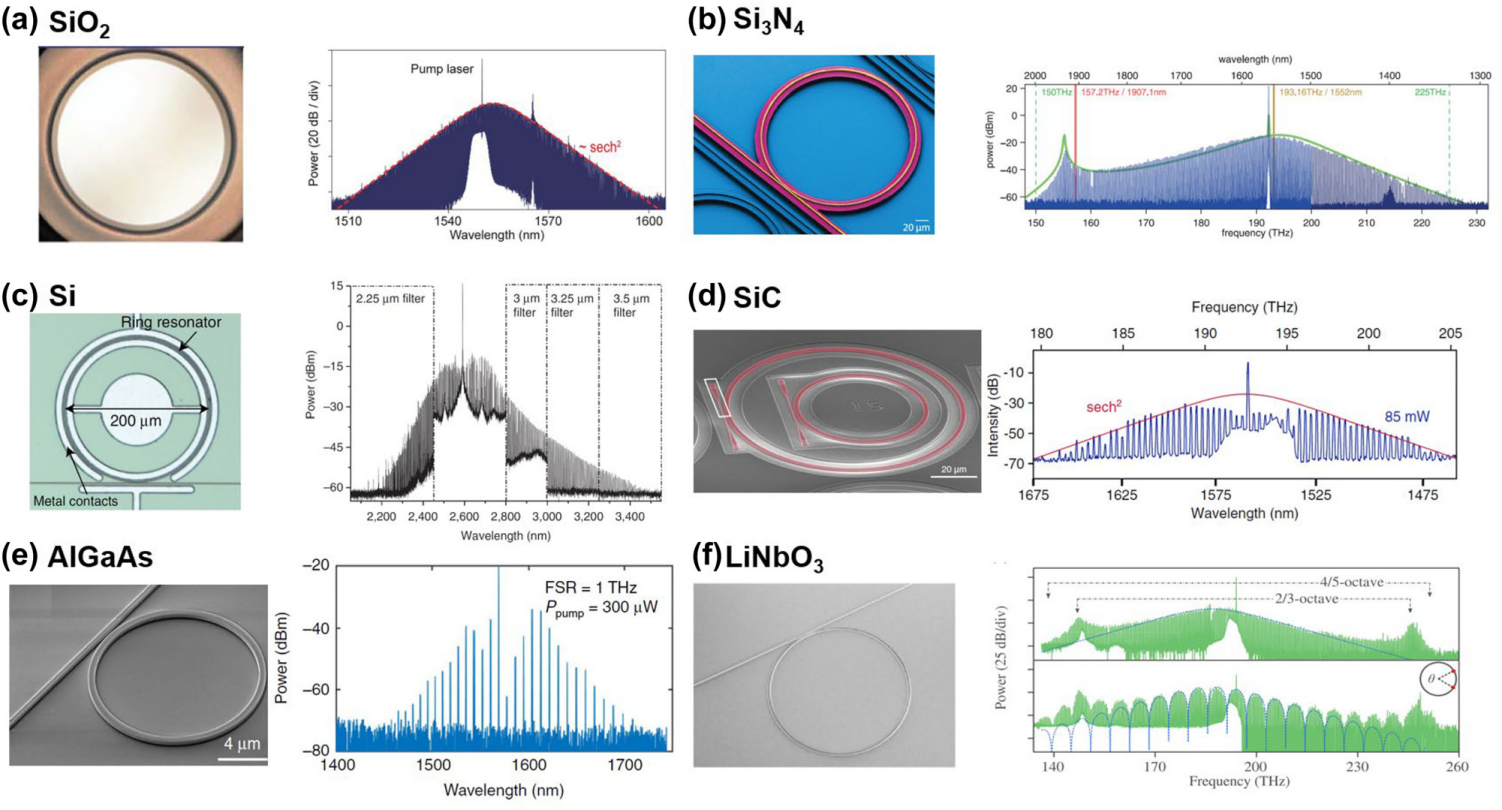
SEM and output spectra of frequency comb based on different integrated platforms: (a) silica wedge resonator [188], (b) silicon nitride microring resonator [174], (c) silicon microring resonator [189], (d) silicon carbide microring resonator [190], (e) AlGaAs microring resonator [192], and (f) lithium niobate microring resonator [193]. (a) Adapted with permission from [188] © Optica Publishing Group. (b) From [174]. Reprinted with permission from AAAS. (c) From [189]. Adapted with permission from Springer Nature. (d) Adapted with permission from [190] © Optica Publishing Group. (e) Adapted from [192] under a CC-BY license. (f) Adapted with permission from [193] © Optica Publishing Group.

Despite the relatively low Kerr coefficient of silica, its low loss and smooth surface enable resonators in the form of microtoroids [159] and microspheres [198] to obtain high quality factors, thereby achieving optical frequency comb generation. To be integrated with the coupler, silica wedge and disk resonators is developed [188], [199], [200]. Yi et al. demonstrated the soliton mode locking in high-*Q* (*Q* ≈ 4 × 10^8^) silica wedge resonator as shown in Figure 10(a). The resonators produce low-phase-noise soliton pulse trains at readily detectable pulse rates. Furthermore, the feedback control is introduced to stabilize the mode-locked system for long time [188]. To improve the compatibility and stability of silica-based microresonators, researchers also introduce high-index doped silica to improve the performance [162], [201]–[204].

Figure 4 illustrates that the transparency range of silicon nitride extends from the visible spectrum to the mid-infrared region, which enables comb generation over a broadband spectrum [163], [165], [178], [205], [206]. Based on a silicon nitride microresonator, a fully coherent microcomb state, spanning 2/3 of an octave, was demonstrated by Brasch et al. [174] through applying the emission of soliton Cherenkov radiation as depicted in Figure 10(b). This design can be self-referenced by doubling and tripling the high and low end of the spectrum, respectively (2*f* − 3*f* technique). The excellent compatibility of silicon nitride enables its applications in large-scale chip integration [207], [208]. The broad transparency range and significant third-order optical nonlinearity make silicon an effective medium for mid-infrared frequency generation. As illustrated in Figure 10(c), an optical frequency comb spans from 2.1 to 3.5 μm with 150 mW pump power [189]. The same team also demonstrated soliton mode-locking comb generation in this microresonator design [209].

In recent years, silicon carbide has become a promising nonlinear photonic material for OFC generation [191], [210] due to its wide transparency window (0.37–5.6 μm [211]), strong X^(2)^ and X^(3)^ nonlinearity, relatively high refractive index (2.6 at 1,550 nm), and compatibility with traditional CMOS processes. Guidry et al. fabricated SiC microring resonators with *Q* = 2.7 × 10^5^, which generated OFC spanning 200 nm in Figure 10(d) [190]. In 2022, Cai et al. demonstrated the first octave-spanning microcomb generation from 1.1 to 2.4 μm in the 4H-SiC platform [210]. 4H-SiC microdisk resonators with a mean *Q* factor up to 6.75 × 10^6^ were fabricated and achieved broadband Kerr frequency combs covering from 1.3 to 1.7 μm when a 13 mW pump was injected [191].

With high nonlinear coefficient, the pump-threshold for comb generation based on AlGaAs is relatively low [192], [212]–[214]. Chang et al. demonstrated a microring resonator in the AlGaAs-on-insulator platform with *Q* beyond 1.5 × 10^6^. The generated comb spectrum with FSR = 1 THz covered 250 nm in Figure 10(e) [192]. The abundant nonlinear optical attributes of lithium niobate significantly enhance the range of functions and applications for combs based on microresonators [215]–[217]. Figure 10(f) shows output spectra of the single-soliton and two-soliton OFC based on LiNbO_3_ ring-shaped microresonator, featuring the repetition rate of 335 GHz and 4/5 octave spanning with two DWs emission [193].

Beyond the materials already mentioned, there are other materials not yet discussed that are used for microresonator-based optical frequency comb generation, such as diamond [194], tantalum pentoxide [195], [218], [219], gallium phosphide [196], and aluminum nitride [197], [220], [221]. Research and application of these materials are continuously evolving, indicating further expansion and deepening of optical frequency comb technology.

### 4.2 SC-based OFC

As depicted in Figure 8(b), spectral broadening is achieved by pumping a nonlinear waveguide with a series of ultrashort pulses with high peak power from an MLL source, which already is an optical frequency comb [48], [112]. The length of waveguide is generally on the order of millimeters. The line spacing of this kind of OFC is determined by the pump laser. Optical coherence covering the entire output spectrum is a key element of the resulting frequency comb. Detailed discussions can be found in [43]. An octave-spanning OFC generated in a silicon waveguide with parameters of SCG in Table 2 is proposed in 2014 [95]. In 2024, Wu et al. combined the *χ* ^(2)^ and *χ* ^(3)^ nonlinearities of LiNbO_3_ in SC generation to achieve gap-free OFC coverage spanning 330–2,400 nm with parameters summarized in Table 2 [104]. Furthermore, coherent supercontinuum generation can be used to further broaden the generated OFC by microresonator in practical applications [222].

### 4.3 EO-based OFC

**Figure 11:**
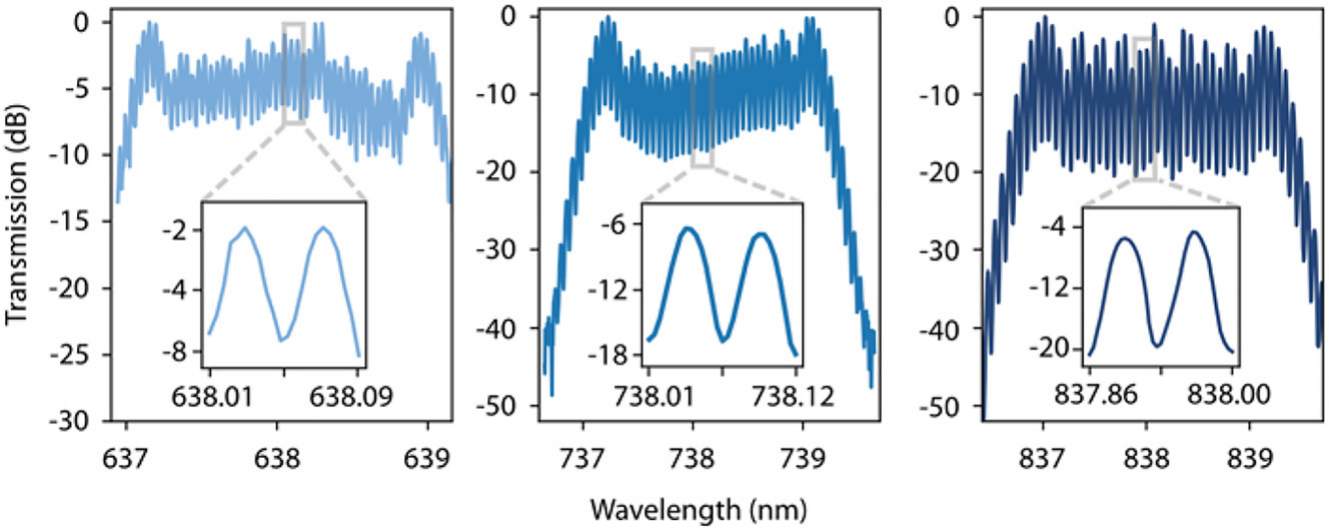
Integrated visible-to-near-infrared electro-optic frequency combs [226]. Adapted from [226] under a CC-BY license.

The electro-optic effect is a nonlinear phenomenon, where the second-order nonlinear process Pockels effect dominates. The Electro-Optic (E-O) comb is generated through the process that cascades sum-frequency generation and difference-frequency generation processes of the pump signal and the modulation signal. Figure 8(c) illustrates that under the modulation of a single-frequency microwave signal, sidebands emerge on both sides of the pump frequency from the continuous wave laser. The conventional and straightforward method for producing EO combs involves directing a continuous wave pump through single or cascaded modulators that are activated by RF sources [223]–[225]. This method offers the advantage of easily selecting and tuning the repetition rate and operational wavelength of the comb as required. Renaud et al. in 2023 demonstrated an integrated and tunable EO frequency comb with over 50 lines in a single comb at 638, 738, and 838 nm and flat-top spectra as shown in Figure 11 [226]. In another type of EO-based OFC, electro-optical phase modulator is positioned within a resonant cavity, where the optical path length is an integral multiple of the pump wavelength. This setup is driven by a microwave signal, whose modulation frequency matches an integral multiple of the FSR in resonator. Through resonance enhancement and frequency conversion processes, optical frequency combs undergo broadening [227]. An EO comb based on LiNbO_3_ thin film microresonators with output spectrum covering 80 nm with beyond 900 separated frequencies spaced is reported by Zhang et al. [228]. In 2020, to improve the efficiency of the cavity-based EO comb, Hu et al. demonstrated an integrated comb covering 132 nm with 30 % pump-to-comb conversion efficiency by applying two mutually coupled resonators [229]. EO-comb can also be used for spectrally tunable dual-comb spectroscopy [230].

### 4.4 MLL-based OFC

**Figure 12:**
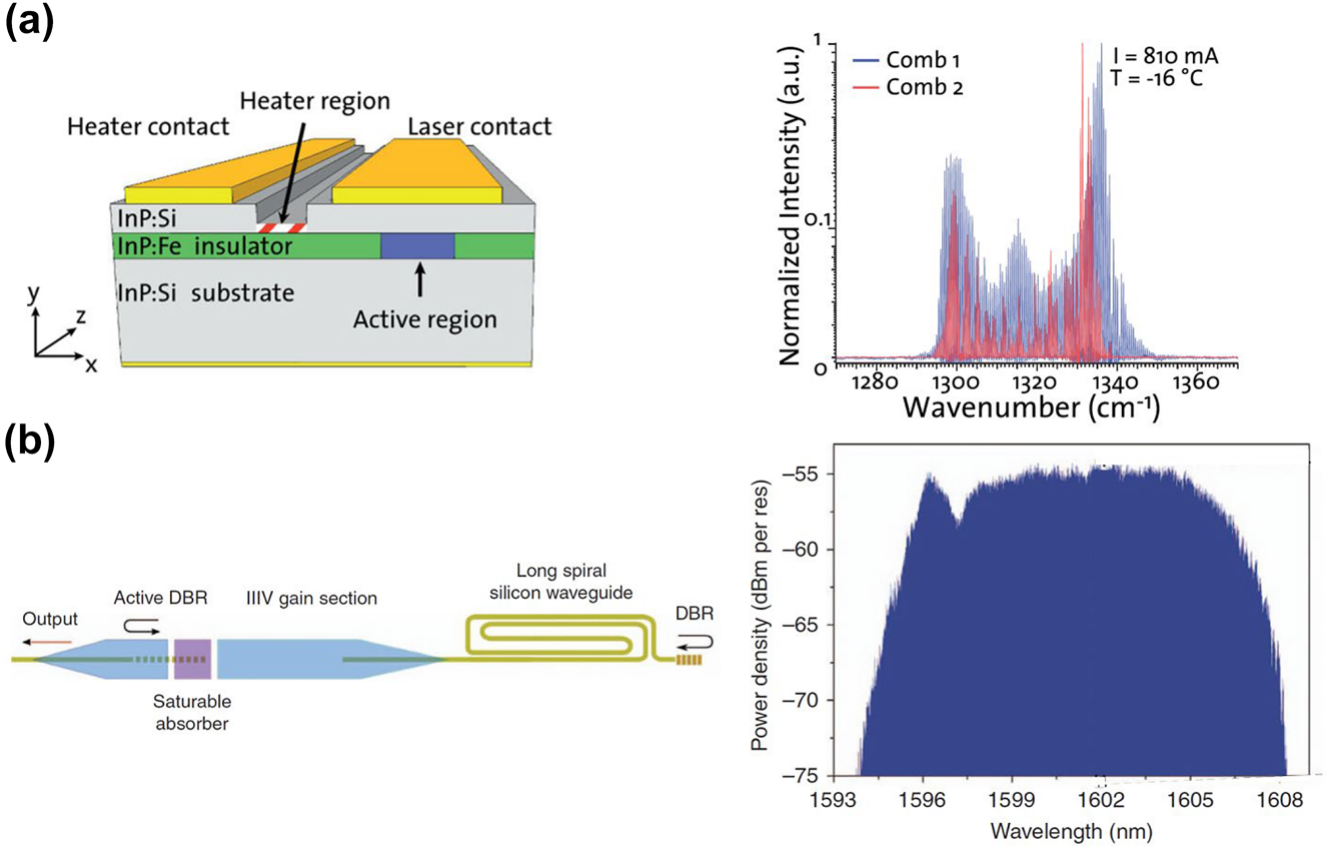
Structures and output spectra of on-chip MLLs. (a) Two combs generated in QCLs [238]. Reprinted from [238], with the permission of AlP Publishing. (b) A III–V-on-Si ultra-dense comb laser [239]. Adapted from [239] under a CC-BY license.

The semiconductor MLL is able to generate a coherent comb with all the phase-locked longitudinal modes and equidistant spacing frequency lines [231]. Figure 9 summarizes the history of MLL for OFC with dark red mark, focusing on the semiconductor MLL with integration potential. The first MLL was reported by Hargrove et al. in 1964 [232]. Ho et al. introduced the active semiconductor MLL in 1978 [233]. Two years later, passive semiconductor MLL was reported by Ippen et al. [234]. To achieve a net increase in gain, in most MLLs, the pulses are passively generated through saturable absorbers or nonlinear elements. Passive mode-locking in quantum dot (QD) laser is observed in 2001 [235]. In 2007, Koch et al. demonstrated a hybrid Si-based quantum-wall (QW) MLL with 40 GHz repetition rate [236]. In 2012, a mid-infrared OFC was generated by a quantum cascade laser (QCL) [237]. In this context, an integrated dual-comb source based on mid-infrared QCL emerged as shown in Figure 12(a) [238]. Through the integration of two independent miniature heaters directly onto each QCL, the fine tuning of the repetition rates and offset frequencies is achievable by exploiting the temperature-dependent tuning of the material refractive index. This setup enables the realization of a precisely controlled dual-comb system. In 2017, Wang et al. demonstrated a heterogeneous ultra-dense comb laser via III–V-on-Si as shown in Figure 12(b) [239]. Locked in the passive mode at 1 GHz repetition rate, the 12-nm output optical spectrum consisted of over 1,400 equidistant frequency lines. In 2020, Meng et al. proposed a mid-infrared OFC from QCL with a buried heterostructure ring cavity. The output spectrum exhibiting a sech^2^ profile [240]. In 2021, ring-type terahertz QCL combs were reported [241]. Dong et al. reported a 60 GHz O-band QD mode-locked laser where the amplitude-modulated and frequency-modulated comb can be generated independently in 2023 [242]. Kazakov et al. reported an active ring resonator based on QCL gain regions that operate in the mid-IR. This device could work as a filter, a wavelength converter, or a frequency comb generator [243].

## 5 Applications

The exponential surge in global data traffic necessitates enhanced processing capabilities within contemporary optical networks. Conventional approaches reliant on electrical signal processing suffer from energy inefficiency stemming from frequent light/electricity and electricity/light conversions. These methods encounter challenges such as low conversion efficiency and speed limitations. All-optical signal processing technology circumvents these issues by directly handling signals in the optical domain, rendering it the preferred solution. Integration of lasers onto chips facilitates the realization of fully integrated optical chips, offering promising prospects for network optimization. OFC provides the link between optical frequency and microwave, making the low noise microwave available.

### 5.1 All-optical switching

Optical switches represent a cornerstone of all-optical signal processing technology, playing a pivotal role in the operational efficacy of both photonic and quantum computing systems. Their performance greatly influences the overall functionality and efficiency of these advanced technological frameworks. Hence, achieving optical switches with high speed, low power consumption, high contrast, and compact size is imperative.

**Figure 13:**
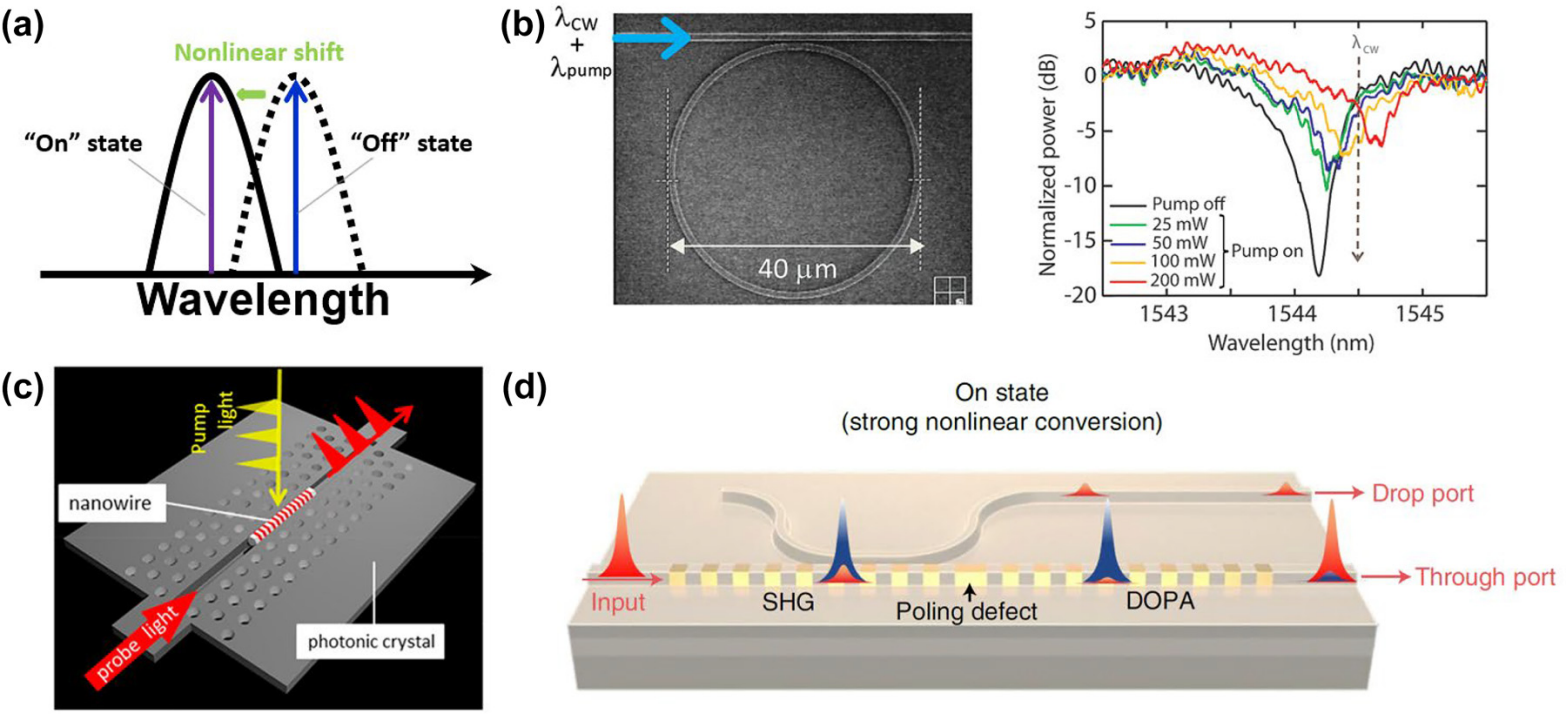
On-chip all-optical switching. (a) Basic concept of all-optical switching. (b) SEM and transmission spectra of a silicon-based ring resonator at different peak levels [245]. Adapted with permission from [245]. Copyright 2010 American Chemical Society. (c) Schematic of a InP/InAsP single-nanowire all-optical switch on a silicon photonic crystal [246]. Adapted with permission from [246]. Copyright 2020 American Chemical Society. (d) Schematic of the lithium niobate nanowaveguides for switching and its operation in the on state when the input pulse energy is high [247]. From [247]. Adapted with permission from Springer Nature.

Based on the third nonlinear optical effects [244], all-optical switching enables the modulation of the signal light propagation by controlling pump light that can induce variations in the refractive index of nonlinear materials, resulting in shifts in the wavelength of the signal light as illustrated in Figure 13(a).

In microring resonators, the change in material refractive index alters the resonance frequency of the microring or the coupling between the microring and the waveguide, thereby modifying the transmittance of the light beam. Martínez et al. proposed a silicon slot ring resonator filled with silicon nanocrystals (Si-nc)/SiO_2_ as a higher Kerr nonlinear medium compared to silicon, to achieve ultrafast all-optical switching on silicon as depicted in Figure 13(b) [245]. A modulation depth over 50 % has been achieved for on-chip optical powers of the order of 100 mW.

Photonic crystal cavities based on thermo-optics and carrier effects are also important devices for realizing all-optical switching. All-optical switching by a subwavelength InP/InAsP nanowire on a silicon photonic crystal was demonstrated by Takiguch et al. as shown in Figure 13(c), which has the 150-ps switching time and a few hundred femtojoules switching energy [246].

In order to achieve lower energy per bit and switching time, *χ* ^(2)^ nonlinear effects have also been proposed to achieve all-optical switching. Guo et al. utilized lithium niobate nanowaveguides in Figure 13(d) for the realization of cavity-free all-optical switching. Through adjusting the dispersion and quasi-phase matching simultaneously, the switching energies could be reduced to 80 fJ with ∼46 fs switching time [247].

The development of all-optical switching is advancing rapidly with the aim to faster response time, reduced power consumption, enhanced switching efficiency, and compact device size. Simultaneously, efforts are made toward ensuring compatibility with CMOS technology. All-optical switching holds immense promise for applications in ultra-high-speed optical interconnection systems, quantum optical chips, and various other domains.

### 5.2 Data transmission

In optical communication systems, high-speed electrical signals are modulated onto optical carriers, utilizing wavelength division multiplexing for parallel data transmission. Chip-based frequency combs, providing numerous equidistant optical carriers, serve as a compact alternative to the extensive laser arrays commonly employed in WDM systems [248], [249]. This innovation facilitates efficient parallel WDM transmission, significantly minimizing both the system’s footprint and energy requirements. In 2014, Pfeifle et al. achieved data rates of 1.44 Tbit/s and spectral efficiencies of 6 bit/s/Hz, marking the first experimental demonstration of coherent data communication utilizing a Kerr comb as the source [250]. To further increase the data rates, the same team used two interleaved dissipative Kerr soliton combs to transmit a data stream of more than 50 Tbit/s on 179 individual optical carriers [207]. In 2018, Hu et al. achieved up to 661 Tbit/s data rates by performing SCG-based comb in an AlGaAsOI-chip waveguide with a single-mode, 30-core fiber [251]. To improve the conversion efficiency of power, Fülöp et al. reported the first coherent WDM transmission experiment conducted with a dark-pulse Kerr comb [252]. The use of combs as sources for wavelength division multiplexing has significantly evolved [253], [254].

**Figure 14:**
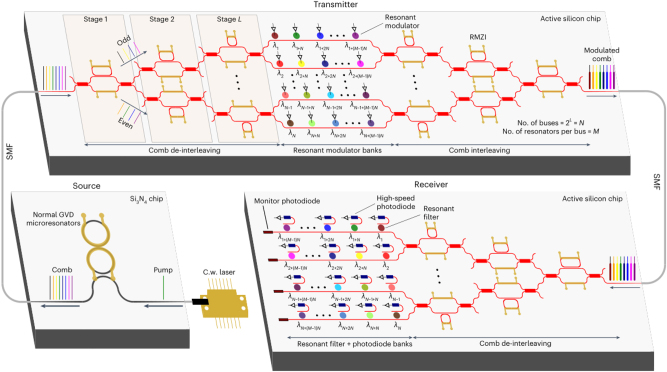
Schematic of the integrated massively scalable silicon photonic data communication link [258]. Adapted from [258] under a CC-BY license.

With the rapid growth of data traffic driven by video services and machine learning, hyperscale data centers and high-performance computers, as primary components of cloud computing, handle the majority of computational workloads [255]. The scale, speed, and energy consumption of interconnections between servers, memory, and computational resources significantly impact the performance. Therefore, chip-based photonic interconnect technology is being introduced into system architectures [137], [138], [256], [257]. Rizzo et al. demonstrated an integrated massively scalable silicon photonic data communication link based on a comb source as shown in Figure 14. Si_3_N_4_ ring resonators were used to generate Kerr comb and then subdivided using tree of standard asymmetric Mach–Zehnder interferometer (MZI). In the transmitter, the subdivided frequency comb was modulated through banks of cascaded microdisk modulators. In the receiver, comb after subdividing was incident on cascaded microring filters with photodiodes. This link architecture demonstrated a data rate of 512 Gb/s across 32 independent wavelength channels [258].

**Figure 15:**
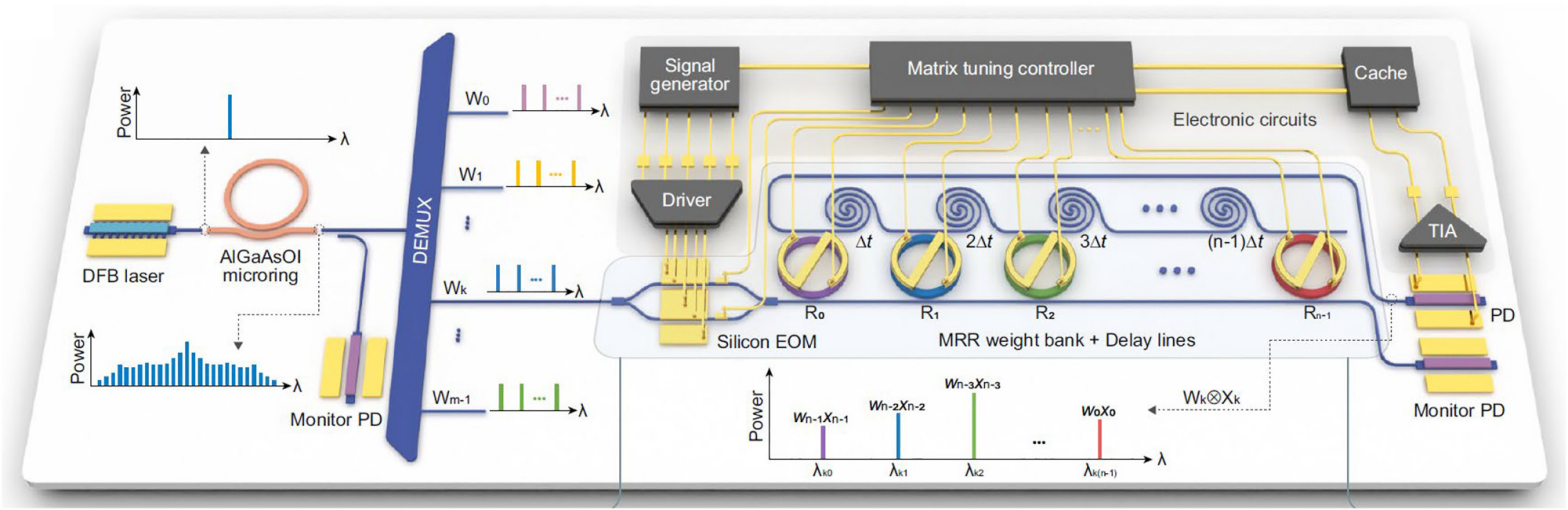
Conceptual drawing of the fully integrated photonic processing unit for optical neural network [259]. Adapted from [259] under a CC-BY license.

### 5.3 Optical neural networks

As an important research object of artificial intelligence, artificial neural network (ANN) establishes connections between neurons in each layer of the neural network by imitating the structure of the nervous system. Optical neural networks (ONNs) emerged as an attractive solution for ANN [260]–[262]. By applying the large number of wavelength channels providing by OFC, the fully integrated ONN is promising. In 2021, Xu et al. proposed a universal optical vector convolutional accelerator operating at 11 TOPS (trillions (10^12^) of operations per second) by interleaving wavelength, temporal and spatial dimensions using a microresonator-based comb [142]. A photonic tensor core for parallel convolutional processing was reported by Feldmann et al. [144], by utilizing the phase-change materials and integrated OFCs. In 2023, Bai et al. demonstrated a photonic processing unit with all the essential photonic components integrated as depicted in Figure 15 [259]. The comb is directly pumped by a chip-based DFB laser and kernel weights in convolutional neural networks are mapped to the microring resonator (MRR) array. This compact architecture enables over 1 TOPS/mm^2^ compute density and competitive edge detection and handwritten digit recognition accuracy (96.6 %). Currently, the majority of on-chip optical devices primarily concentrate on the linear operations of convolutional neural network [208], [263]–[265]. Further exploration into implementing nonlinear operations on integrated optical chips is still warranted.

### 5.4 Microwave signal generation

**Figure 16:**
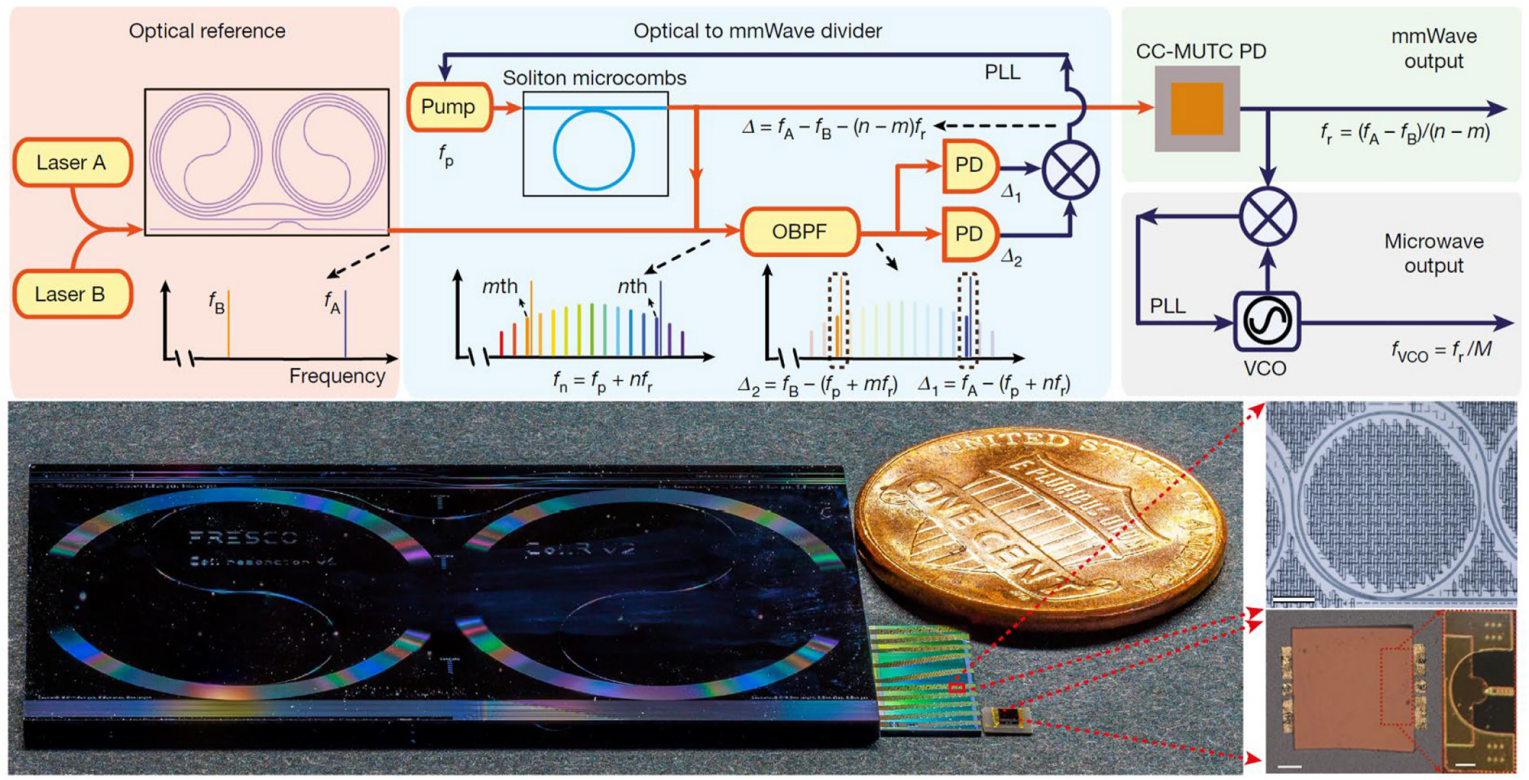
Schematic and photograph of critical elements of integrated OFD [268]. Adapted from [268] under a CC-BY license.

The microwave source with low-noise and high spectral purity is of vital important in various fields, including communications, positioning, metrology and spectroscopy. Optical Frequency Division (OFD) enables the frequency down-conversion of ultra-stable optical references, facilitating the generation of microwaves. Two-point locking with a frequency comb is a preferable method [266], [267]. Different from the previous bulk or fiber-based OFD systems, several research groups unveiled on-chip integrated photonics OFD in early 2024, showcasing the generation of low-noise microwaves with compact platforms. Sun et al. demonstrated a miniaturized OFD system with the potential to be applied to a CMOS platform as shown in Figure 16 [268]. Two commercial semiconductor lasers are frequency stabilized to an integrated coil cavity to serve as the optical reference. The frequency difference of the two reference lasers is divided down to mmWave by an OFC. The generated signal at 100 GHz reaches a phase noise of −114 dBc/Hz at 10 kHz offset frequency. Kudelin et al. prestabilized the outputs of two DFB lasers via self-injection locked into spiral resonators and then locked them to a miniature Fabry–Pérot cavity. The generated microwave signal is at 20 GHz with phase noise of −135 dBc/Hz at 10 kHz offset [269]. Zhao et al. proposed an all-optical OFD on a chip using a single laser with the scaled phase noise of −128 dBc/Hz for the 16 GHz devices [270]. Two Kerr microresonators under distinct dynamical states are synchronized, where the inherent stability of the terahertz beat frequency between the signal and idler fields of an optical parametric oscillator is transferred to a microwave frequency of a Kerr soliton comb. The breakthrough of OFD paves the way for the development of compact, portable, scalable, and cost-effective microwave synthesis solutions tailored for a range of critical commercial applications, including navigation, communications, and precise timing.

### 5.5 Quantum information processing

**Figure 17:**
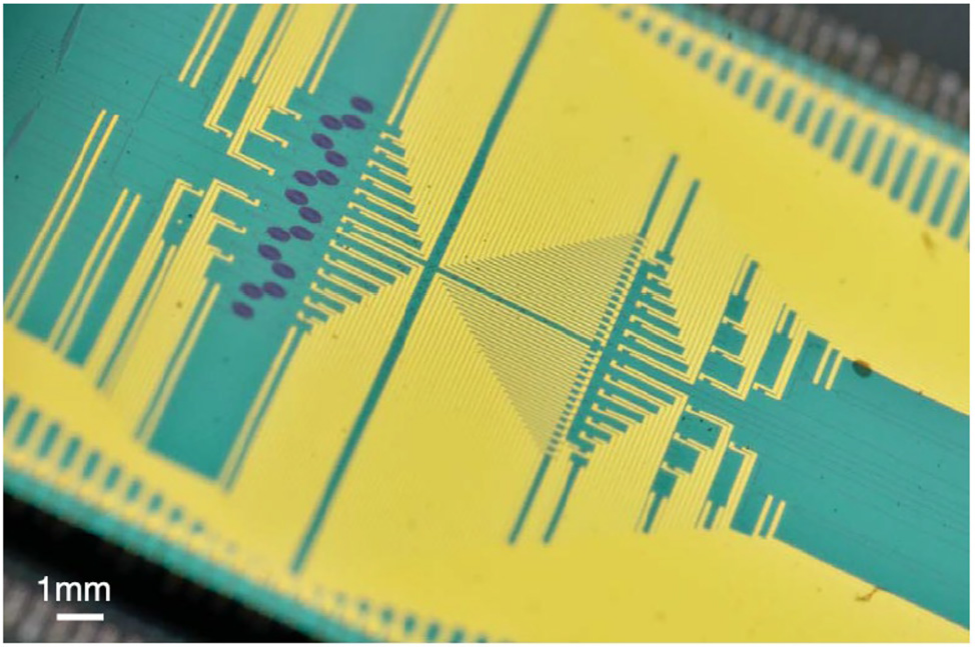
Photograph of a multidimensional integrated quantum photonic platform [277]. From [277]. Reprinted with permission from AAAS.

Based on the fundamental properties of quantum mechanics such as superposition and entanglement, quantum information processing surpasses classical technologies in communication, precision measurement, computation, and simulation. In recent years, integrated photonic quantum chips have emerged as a critical means of realizing quantum information processing, due to their advantages of small size, high precision, good stability, and strong scalability [271]. Single-photon sources (SPDs), single-photon detectors, quantum switching, and manipulation devices are looking forward to be integrated in a chip. Pumping nonlinear waveguides or microcavities could generate photon pairs through the process of spontaneous four-wave mixing (SFWM) [272]–[274] or spontaneous parametric down conversion (SPDC) [275], [276]. By using one of the photons as a predictor, a single photon source can be realized. In 2018, Wang et al. demonstrated a multidimensional integrated quantum photonic platform with beyond 550 photonic components as shown in Figure 17 [277]. This chip including 16 identical photon-pair sources could generate, control, and analyze high-dimensional entanglement. Based on advanced CMOS manufacturing processes and integrating optical elements manufactured from various material systems [278], photonic quantum chips have made great progress in the past decade [279]. However, the integration of light sources and detectors into a system still remain to be addressed [280].

## 6 Conclusion and outlook

This review summarizes the advancements and applications of integrated nonlinear photonics, spotlighting the SC and OFC generation. The recent applications of chip-based nonlinear photonics are introduced. The silicon waveguide remains paramount in nonlinear photonics due to its cost-effective high-level integration afforded by established silicon circuitry techniques. Si_3_N_4_ and chalcogenide waveguides are notable for their versatility across various nonlinear domains. The novel material platforms with high nonlinearity and low loss also emerge, further expanding the working range of SC and OFC. Progress in material synthesis, component construction, and novel strategies for enabling nonlinear phenomena with energy-efficient devices is sparking innovations across diverse domains. Although integrated nonlinear photonics have achieved considerable milestones on a single device level, weaving them into fully integrated photonic systems remains an area ripe for further exploration. For the functioning of these systems, substantial electronic and optical components are currently required. To minimize the size, weight and power consumption, and cost (SWaP-C) in commercial applications, it’s essential to integrate sources with segments of the optical systems in photonic integrated circuits [62]. Emerging hybrid and heterogeneous integrations have the potential to combine the advancement of various technologies on a single platform. Such endeavors are fast-tracking numerous system-level solutions, including optical-frequency synthesizers [134], optical atomic clocks [133], and optical signal processing [281]. Over recent decades, the field has navigated through numerous technological and manufacturing hurdles. During this rapid progression, there is immense anticipation for the realization of large-scale integrated nonlinear photonics, expected to spark a new era of innovative technologies in fields such as data communication, ranging, spectroscopy, optical neural networks, and quantum optics.

## References

[1] Franken P. A., Hill A. E., Peters C. E., Weinreich G. (1961). Generation of optical harmonics. *Phys. Rev. Lett.*.

[2] Boyd R. W., Gaeta A. L., Giese E. (2023). *Springer Handbook of Atomic, Molecular, and Optical Physics*.

[3] Malvezzi A. (2003). Resonant second-harmonic generation in a GaAs photonic crystal waveguide. *Phys. Rev. B*.

[4] Billat A., Grassani D., Pfeiffer M. H., Kharitonov S., Kippenberg T. J., Brès C.-S. (2017). Large second harmonic generation enhancement in Si_3_N_4_ waveguides by all-optically induced quasi-phase-matching. *Nat. Commun.*.

[5] Shen Y. R. (1989). Surface properties probed by second-harmonic and sum-frequency generation. *Nature*.

[6] Lambert A. G., Davies P. B., Neivandt D. J. (2005). Implementing the theory of sum frequency generation vibrational spectroscopy: a tutorial review. *Appl. Spectrosc. Rev.*.

[7] Morita A., Hynes J. T. (2000). A theoretical analysis of the sum frequency generation spectrum of the water surface. *Chem. Phys.*.

[8] Erny C. (2007). Mid-infrared difference-frequency generation of ultrashort pulses tunable between 3.2 and 4.8 μm from a compact fiber source. *Opt. Lett.*.

[9] Belkin M. A. (2008). Room temperature terahertz quantum cascade laser source based on intracavity difference-frequency generation. *Appl. Phys. Lett.*.

[10] Belkin M. A. (2007). Terahertz quantum-cascade-laser source based on intracavity difference-frequency generation. *Nat. Photonics*.

[11] Leidinger M., Fieberg S., Waasem N., Kühnemann F., Buse K., Breunig I. (2015). Comparative study on three highly sensitive absorption measurement techniques characterizing lithium niobate over its entire transparent spectral range. *Opt. Express*.

[12] Liu X. (2019). Beyond 100 THz-spanning ultraviolet frequency combs in a non-centrosymmetric crystalline waveguide. *Nat. Commun.*.

[13] Aitchison J. S., Hutchings D., Kang J., Stegeman G., Villeneuve A. (1997). The nonlinear optical properties of AlGaAs at the half band gap. *IEEE J. Quantum Electron.*.

[14] Lin Y.-Y. (2016). Self-phase modulation in highly confined submicron Ta_2_O_5_ channel waveguides. *Opt. Express*.

[15] Hsieh I. W. (2006). Ultrafast-pulse self-phase modulation and third-order dispersion in Si photonic wire-waveguides. *Opt. Express*.

[16] Liu X. (2011). Self-phase modulation and nonlinear loss in silicon nanophotonic wires near the mid-infrared two-photon absorption edge. *Opt. Express*.

[17] Dulkeith E., Vlasov Y. A., Chen X., Panoiu N. C., Osgood R. M. (2006). Self-phase-modulation in submicron silicon-on-insulator photonic wires. *Opt. Express*.

[18] Dumais P. (1993). Enhanced self-phase modulation in tapered fibers. *Opt. Lett.*.

[19] Wang Z., Liu H., Huang N., Sun Q., Wen J. (2012). Efficient terahertz-wave generation via four-wave mixing in silicon membrane waveguides. *Opt. Express*.

[20] Yang Y. (2018). High-efficiency and broadband four-wave mixing in a silicon-graphene strip waveguide with a windowed silica top layer. *Photonics Res.*.

[21] Fukuda H. (2005). Four-wave mixing in silicon wire waveguides. *Opt. Express*.

[22] Raghunathan V., Claps R., Dimitropoulos D., Jalali B. (2004). Wavelength conversion in silicon using Raman induced four-wave mixing. *Appl. Phys. Lett.*.

[23] Wathen J. J., Apiratikul P., Richardson C. J. K., Porkolab G. A., Carter G. M., Murphy T. E. (2014). Efficient continuous-wave four-wave mixing in bandgap-engineered AlGaAs waveguides. *Opt. Lett.*.

[24] Hsieh I. W. (2007). Cross-phase modulation-induced spectral and temporal effects on co- propagating femtosecond pulses in silicon photonic wires. *Opt. Express*.

[25] Genty G., Lehtonen M., Ludvigsen H. (2004). Effect of cross-phase modulation on supercontinuum generated in microstructured fibers with sub-30 fs pulses. *Opt. Express*.

[26] Boyraz Ö., Koonath P., Raghunathan V., Jalali B. (2004). All optical switching and continuum generation in silicon waveguides. *Opt. Express*.

[27] Fischer M. P. (2018). Plasmonic mid-infrared third harmonic generation in germanium nanoantennas. *Light: Sci. Appl.*.

[28] Wang C. C., Bomback J., Donlon W. T., Huo C. R., James J. V. (1986). Optical third-harmonic generation in reflection from crystalline and amorphous samples of silicon. *Phys. Rev. Lett.*.

[29] Burns W. K., Bloembergen N. (1971). Third-harmonic generation in absorbing media of cubic or isotropic symmetry. *Phys. Rev. B*.

[30] Levy J. S., Foster M. A., Gaeta A. L., Lipson M. (2011). Harmonic generation in silicon nitride ring resonators. *Opt. Express*.

[31] Bristow A. D., Rotenberg N., van Driel H. M. (2007). Two-photon absorption and Kerr coefficients of silicon for 850-2200 nm. *Appl. Phys. Lett.*.

[32] Leuthold J., Koos C., Freude W. (2010). Nonlinear silicon photonics. *Nat. Photonics*.

[33] Foster M. A., Turner A. C., Sharping J. E., Schmidt B. S., Lipson M., Gaeta A. L. (2006). Broad-band optical parametric gain on a silicon photonic chip. *Nature*.

[34] Da Ros F. (2018). Dual-polarization wavelength conversion of 16-QAM signals in a single silicon waveguide with a lateral p-i-n diode [Invited]. *Photonics Res.*.

[35] Joshi C. (2020). Frequency-domain quantum interference with correlated photons from an integrated microresonator. *Phys. Rev. Lett.*.

[36] Vermeulen N. (2023). Post-2000 nonlinear optical materials and measurements: data tables and best practices. *J. Phys.: Photonics*.

[37] Kasap S., Capper P. (2006). *Springer Handbook of Electronic and Photonic Materials*.

[38] Zhang L., Agarwal A. M., Kimerling L. C., Michel J. (2014). Nonlinear Group IV photonics based on silicon and germanium: from near-infrared to mid-infrared. *Nanophotonics*.

[39] Harris D. C. (1998). Durable 3-5 μm transmitting infrared window materials. *Infrared Phys. Technol.*.

[40] Palik E. D. (1998). *Handbook of Optical Constants of Solids*.

[41] Hon N. K., Soref R., Jalali B. (2011). The third-order nonlinear optical coefficients of Si, Ge, and Si_1−x_Ge_x_ in the midwave and longwave infrared. *J. Appl. Phys.*.

[42] Miyazaki S., Nishimura H., Fukuda M., Ley L., Ristein J. (1997). Structure and electronic states of ultrathin SiO_2_ thermally grown on Si (100) and Si (111) surfaces. *Appl. Surf. Sci.*.

[43] Gaeta A. L., Lipson M., Kippenberg T. J. (2019). Photonic-chip-based frequency combs. *Nat. Photonics*.

[44] Soref R. A., Emelett S. J., Buchwald W. R. (2006). Silicon waveguided components for the long-wave infrared region. *J. Opt. A: Pure Appl. Opt.*.

[45] Malitson I. H. (1965). Interspecimen comparison of the refractive index of fused silica. *J. Opt. Soc. Am. B*.

[46] Tan D. T. H., Ikeda K., Sun P. C., Fainman Y. (2010). Group velocity dispersion and self phase modulation in silicon nitride waveguides. *Appl. Phys. Lett.*.

[47] Moss D. J., Morandotti R., Gaeta A. L., Lipson M. (2013). New CMOS-compatible platforms based on silicon nitride and Hydex for nonlinear optics. *Nat. Photonics*.

[48] Guo H. (2018). Mid-infrared frequency comb via coherent dispersive wave generation in silicon nitride nanophotonic waveguides. *Nat. Photonics*.

[49] Philipp H. R. (1973). Optical properties of silicon nitride. *J. Electrochem. Soc.*.

[50] Ikeda K., Saperstein R. E., Alic N., Fainman Y. (2008). Thermal and Kerr nonlinear properties of plasma-deposited silicon nitride/silicon dioxide waveguides. *Opt. Express*.

[51] Soref R. (2010). Mid-infrared photonics in silicon and germanium. *Nat. Photonics*.

[52] Barnes N. P., Piltch M. S. (1979). Temperature-dependent Sellmeier coefficients and nonlinear optics average power limit for germanium. *J. Opt. Soc. Am*.

[53] Sinobad M. (2020). Mid-infrared supercontinuum generation in silicon-germanium all-normal dispersion waveguides. *Opt. Lett.*.

[54] Laniel J. M., Hô N., Vallée R., Villeneuve A. (2005). Nonlinear-refractive-index measurement in As_2_S_3_ channel waveguides by asymmetric self-phase modulation. *JOSA B*.

[55] Sanghera J. S., Shaw L. B., Aggarwal I. D. (2002). Applications of chalcogenide glass optical fibers. *C. R. Chim.*.

[56] Rodney W. S., Malitson I. H., King T. A. (1958). Refractive index of arsenic trisulfide. *J. Opt. Soc. Am*.

[57] Ogusu K., Shinkawa K. (2009). Optical nonlinearities in As_2_Se_3_ chalcogenide glasses doped with Cu and Ag for pulse durations on the order of nanoseconds. *Opt. Express*.

[58] Mamoun S., Merad A., Guilbert L. (2013). Energy band gap and optical properties of lithium niobate from ab initio calculations. *Comput. Mater. Sci.*.

[59] Zhu D. (2021). Integrated photonics on thin-film lithium niobate. *Adv. Opt. Photonics Res.*.

[60] Villeneuve A., Yang C., Stegeman G. I., Lin C. H., Lin H. H. (1993). Nonlinear refractive‐index and two photon‐absorption near half the band gap in AlGaAs. *Appl. Phys. Lett.*.

[61] Vyas K. (2022). Group III-V semiconductors as promising nonlinear integrated photonic platforms. *Adv. Phys.: X*.

[62] Chang L., Liu S., Bowers J. E. J. N. P. (2022). Integrated optical frequency comb technologies. *Nat. Photonics*.

[63] Taghavi I. (2022). Polymer modulators in silicon photonics: review and projections. *Nanophotonics*.

[64] Wang M. (2023). Perspectives of thin-film lithium niobate and electro-optic polymers for high-performance electro-optic modulation. *J. Mater. Chem. C*.

[65] Alloatti L. (2014). 100 GHz silicon–organic hybrid modulator. *Light: Sci. Appl.*.

[66] Pan Z. (2018). High-speed modulator based on electro-optic polymer infiltrated subwavelength grating waveguide ring resonator. *Laser Photonics Rev.*.

[67] Fang Y. (2023). Recent progress of supercontinuum generation in nanophotonic waveguides. *Laser Photonics Rev.*.

[68] Carlson D. R. (2017). Photonic-chip supercontinuum with tailored spectra for counting optical frequencies. *Phys. Rev. Appl.*.

[69] Nakasyotani T., Toda H., Kuri T., Kitayama K. (2006). Wavelength-division-multiplexed millimeter-waveband radio-on-fiber system using a supercontinuum light source. *J. Lightwave Technol.*.

[70] Zeylikovich I., Kartazaev V., Alfano R. R. (2005). Spectral, temporal, and coherence properties of supercontinuum generation in microstructure fiber. *J. Opt. Soc. Am. B*.

[71] Morioka T., Mori K., Saruwatari M. (1993). More than 100-wavelength-channel picosecond optical pulse generation from single laser source using supercontinuum in optical fibres. *Electron. Lett.*.

[72] Wang K.-Y., Petrillo K. G., Foster M. A., Foster A. C. (2012). Ultralow-power all-optical processing of high-speed data signals in deposited silicon waveguides. *Opt. Express*.

[73] Choi J. W. (2021). High spectro-temporal compression on a nonlinear CMOS-chip. *Light: Sci. Appl.*.

[74] Heidt A. M. (2011). High quality sub-two cycle pulses from compression of supercontinuum generated in all-normal dispersion photonic crystal fiber. *Opt. Express*.

[75] Mollenauer L. F., Stolen R. H., Gordon J. P., Tomlinson W. J. (1983). Extreme picosecond pulse narrowing by means of soliton effect in single-mode optical fibers. *Opt. Lett.*.

[76] Nishizawa N., Chen Y., Hsiung P., Ippen E., Fujimoto J. (2004). Real-time, ultrahigh-resolution, optical coherence tomography with an all-fiber, femtosecond fiber laser continuum at 1.5 µm. *Opt. Lett.*.

[77] Hartl I. (2001). Ultrahigh-resolution optical coherence tomography using continuum generation in an air-silica microstructure optical fiber. *Opt. Lett.*.

[78] Humbert G. (2006). Supercontinuum generation system for optical coherence tomography based on tapered photonic crystal fibre. *Opt. Express*.

[79] Drexler W. (2004). Ultrahigh-resolution optical coherence tomography. *J. Biomed. Opt.*.

[80] Ji X., Mojahed D., Okawachi Y., Gaeta A. L., Hendon C. P., Lipson M. (2021). Millimeter-scale chip-based supercontinuum generation for optical coherence tomography. *Sci. Adv.*.

[81] Dasa M. K., Markos C., Janting J., Bang O. (2019). Multispectral photoacoustic sensing for accurate glucose monitoring using a supercontinuum laser. *J. Opt. Soc. Am. B*.

[82] Yesilkoy F. J. S. (2019). Optical interrogation techniques for nanophotonic biochemical sensors. *Sensors*.

[83] Sieger M., Mizaikoff B. (2016). Toward on-chip mid-infrared sensors. *Anal. Chem.*.

[84] Du Q. (2018). Chip-scale broadband spectroscopic chemical sensing using an integrated supercontinuum source in a chalcogenide glass waveguide. *Photonics Res.*.

[85] Mizaikoff B. (2013). Waveguide-enhanced mid-infrared chem/bio sensors. *Chem. Soc. Rev.*.

[86] Jahromi K. E. (2019). Mid-infrared supercontinuum-based upconversion detection for trace gas sensing. *Opt. Express*.

[87] Luzinova Y., Zdyrko B., Luzinov I., Mizaikoff B. (2012). In situ trace analysis of oil in water with mid-infrared fiberoptic chemical sensors. *Anal. Chem.*.

[88] Lindfors K., Kalkbrenner T., Stoller P., Sandoghdar V. (2004). Detection and spectroscopy of gold nanoparticles using supercontinuum white light confocal microscopy. *Phys. Rev. Lett.*.

[89] Diddams S. A., Hollberg L., Mbele V. (2007). Molecular fingerprinting with the resolved modes of a femtosecond laser frequency comb. *Nature*.

[90] Petersen C. R. (2014). Mid-infrared supercontinuum covering the 1.4-13.3 μm molecular fingerprint region using ultra-high NA chalcogenide step-index fibre. *Nat. Photonics*.

[91] Mak K. F., Ju L., Wang F., Heinz T. F. (2012). Optical spectroscopy of graphene: from the far infrared to the ultraviolet. *Solid State Commun*..

[92] Picqué N., Hänsch T. W. (2019). Frequency comb spectroscopy. *Nat. Photonics*.

[93] Thorpe M. J., Moll K. D., Jones R. J., Safdi B., Ye J. (2006). Broadband cavity ringdown spectroscopy for sensitive and rapid molecular detection. *Science*.

[94] Kou R. (2018). Mid-IR broadband supercontinuum generation from a suspended silicon waveguide. *Opt. Lett.*.

[95] Kuyken B. (2015). An octave-spanning mid-infrared frequency comb generated in a silicon nanophotonic wire waveguide. *Nat. Commun.*.

[96] Singh N. (2015). Midinfrared supercontinuum generation from 2 to 6 μm in a silicon nanowire. *Optica*.

[97] Yoon Oh D., Yang K. Y., Fredrick C., Ycas G., Diddams S. A., Vahala K. J. (2017). Coherent ultra-violet to near-infrared generation in silica ridge waveguides. *Nat. Commun.*.

[98] Brès C.-S., Della Torre A., Grassani D., Brasch V., Grillet C., Monat C. (2023). Supercontinuum in integrated photonics: generation, applications, challenges, and perspectives. *Nanophotonics*.

[99] Della Torre A. (2021). Mid-infrared supercontinuum generation in a low-loss germanium-on-silicon waveguide. *APL Photonics*.

[100] Torre A. D. (2023). Mid-infrared supercontinuum generation in a varying dispersion waveguide for multi-species gas spectroscopy. *IEEE J. Sel. Top. Quantum Electron.*.

[101] Zhao P., Yang L., Xia K., Yang P., Wang R., Xu P. Exceeding two octave-spanning supercontinuum generation in integrated As_2_S_3_ waveguides pumped by a 2 μm fiber laser. *Opt. Laser Technol.*.

[102] Saini T. S., Tiwari U. K., Sinha R. K. (2018). Design and analysis of dispersion engineered rib waveguides for on-chip mid-infrared supercontinuum. *J. Lightwave Technol.*.

[103] Lu J., Surya J. B., Liu X., Xu Y., Tang H. X. (2019). Octave-spanning supercontinuum generation in nanoscale lithium niobate waveguides. *Opt. Lett.*.

[104] Wu T.-H. (2024). Visible-to-ultraviolet frequency comb generation in lithium niobate nanophotonic waveguides. *Nat. Photonics*.

[105] Kuyken B., Billet M., Leo F., Yvind K., Pu M. (2020). Octave-spanning coherent supercontinuum generation in an AlGaAs-on-insulator waveguide. *Opt. Lett.*.

[106] Hsieh I.-W. (2007). Supercontinuum generation in silicon photonic wires. *Opt. Express*.

[107] Lafforgue C. (2022). Supercontinuum generation in silicon photonics platforms. *Photonics Res.*.

[108] Lau R. K. W., Lamont M. R. E., Griffith A. G., Okawachi Y., Lipson M., Gaeta A. L. (2014). Octave-spanning mid-infrared supercontinuum generation in silicon nanowaveguides. *Opt. Lett.*.

[109] Singh N. (2018). Octave-spanning coherent supercontinuum generation in silicon on insulator from 1.06 μm to beyond 2.4 μm. *Light: Sci. Appl.*.

[110] Nader N. (2018). Versatile silicon-waveguide supercontinuum for coherent mid-infrared spectroscopy. *APL Photonics*.

[111] De Leonardis F., Troia B., Soref R. A., Passaro V. M. N. (2015). Modelling of supercontinuum generation in the germanium-on-silicon waveguided platform. *J. Lightwave Technol.*.

[112] Yuan J. (2017). Mid-infrared octave-spanning supercontinuum and frequency comb generation in a suspended germanium-membrane ridge waveguide. *J. Lightwave Technol.*.

[113] Baboux F., Moody G., Ducci S. (2023). Nonlinear integrated quantum photonics with AlGaAs. *Optica*.

[114] Chiles J. (2019). Multifunctional integrated photonics in the mid-infrared with suspended AlGaAs on silicon. *Optica*.

[115] Halir R., Okawachi Y., Levy J. S., Foster M. A., Lipson M., Gaeta A. L. (2012). Ultrabroadband supercontinuum generation in a CMOS-compatible platform. *Opt. Lett.*.

[116] Fang Y. (2020). Three-octave supercontinuum generation using SiO_2_ cladded Si_3_N_4_ slot waveguide with all-normal dispersion. *J. Lightwave Technol.*.

[117] Fang Y., Bao C., Wang Z., Zhang W., Pan Z., Yue Y. (2022). Multiple coherent dispersive waves generation in silicon nitride slot waveguide. *Appl. Phys. Lett.*.

[118] Rebolledo-Salgado I. (2022). Coherent supercontinuum generation in all-normal dispersion Si_3_N_4_ waveguides. *Opt. Express*.

[119] Zhao H. (2015). Visible-to-near-infrared octave spanning supercontinuum generation in a silicon nitride waveguide. *Opt. Lett.*.

[120] Epping J. P. (2015). On-chip visible-to-infrared supercontinuum generation with more than 495 THz spectral bandwidth. *Opt. Express*.

[121] Reig Escalé M., Kaufmann F., Jiang H., Pohl D., Grange R. (2020). Generation of 280 THz-spanning near-ultraviolet light in lithium niobate-on-insulator waveguides with sub-100 pJ pulses. *APL Photonics*.

[122] Jankowski M. (2020). Ultrabroadband nonlinear optics in nanophotonic periodically poled lithium niobate waveguides. *Optica*.

[123] Sinobad M. (2018). Mid-infrared octave spanning supercontinuum generation to 8.5 μm in silicon-germanium waveguides. *Optica*.

[124] Lamont M. R., Luther-Davies B., Choi D.-Y., Madden S., Eggleton B. J. (2008). Supercontinuum generation in dispersion engineered highly nonlinear (*γ*=10/W/m) As_2_S_3_ chalcogenide planar waveguide. *Opt. Express*.

[125] Dave U. D. (2015). Dispersive-wave-based octave-spanning supercontinuum generation in InGaP membrane waveguides on a silicon substrate. *Opt. Lett.*.

[126] Yu Y. (2016). Experimental demonstration of linearly polarized 2-10 μm supercontinuum generation in a chalcogenide rib waveguide. *Opt. Lett.*.

[127] Choi J. W. (2016). Nonlinear characterization of GeSbS chalcogenide glass waveguides. *Sci. Rep.*.

[128] Udem T., Reichert J., Holzwarth R., Hänsch T. (1999). Absolute optical frequency measurement of the cesium D_1_ line with a mode-locked laser. *Phys. Rev. Lett.*.

[129] Jones D. J. (2000). Carrier-envelope phase control of femtosecond mode-locked lasers and direct optical frequency synthesis. *Science*.

[130] Diddams S. A. (2000). Direct link between microwave and optical frequencies with a 300 THz femtosecond laser comb. *Phys. Rev. Lett.*.

[131] Diddams S. A. (2010). The evolving optical frequency comb. *JOSA B*.

[132] Papp S. B. (2014). Microresonator frequency comb optical clock. *Optica*.

[133] Newman Z. L. (2019). Architecture for the photonic integration of an optical atomic clock. *Optica*.

[134] Spencer D. T. (2018). An optical-frequency synthesizer using integrated photonics. *Nature*.

[135] Black J. A., Yu S., Streater R., Stone J. R., Lu X., Moille G., Srinivasan K., Papp S. B. Optical synthesis by spectral translation. *Conference on Lasers and Electro-Optics*.

[136] Yao Y. (2021). Optical frequency synthesizer referenced to an ytterbium optical clock. *Photonics Res.*.

[137] Shu H. (2022). Microcomb-driven silicon photonic systems. *Nature*.

[138] Yang K. Y. (2022). Multi-dimensional data transmission using inverse-designed silicon photonics and microcombs. *Nat. Commun.*.

[139] Suh M.-G. (2016). Microresonator soliton dual-comb spectroscopy. *Science*.

[140] Dutt A. (2018). On-chip dual-comb source for spectroscopy. *Sci. Adv.*.

[141] Yu M., Okawachi Y., Griffith A. G., Picqué N., Lipson M., Gaeta A. L. (2018). Silicon-chip-based mid-infrared dual-comb spectroscopy. *Nat. Commun.*.

[142] Xu X. (2021). 11 TOPS photonic convolutional accelerator for optical neural networks. *Nature*.

[143] Guo X., Xiang J., Zhang Y., Su Y. (2021). Integrated neuromorphic photonics: synapses, neurons, and neural networks. *Adv. Photonics Res.*.

[144] Feldmann J. (2021). Parallel convolutional processing using an integrated photonic tensor core. *Nature*.

[145] Ma B., Zhang J., Zou W. (2022). Comb-based photonic neural population for parallel and nonlinear processing. *Photonics Res.*.

[146] Willner A. E., Fallahpour A., Zou K., Alishahi F., Zhou H. (2020). Optical signal processing aided by optical frequency combs. *IEEE J. Sel. Top. Quantum Electron.*.

[147] Moss D. (2021). Ultra-high bandwidth radio frequency and microwave photonic signal processing based on kerr micro-combs. *Adv. Phys.: X*.

[148] Riemensberger J. (2020). Massively parallel coherent laser ranging using a soliton microcomb. *Nature*.

[149] Guidry M. A., Lukin D. M., Yang K. Y., Trivedi R., Vučković J. (2022). Quantum optics of soliton microcombs. *Nat. Photonics*.

[150] Lu H.-H., Liscidini M., Gaeta A. L., Weiner A. M., Lukens J. M. (2023). Frequency-bin photonic quantum information. *Optica*.

[151] Mahmudlu H. (2023). Fully on-chip photonic turnkey quantum source for entangled qubit/qudit state generation. *Nat. Photonics*.

[152] Braginsky V. B., Gorodetsky M. L., Ilchenko V. S. (1989). Quality-factor and nonlinear properties of optical whispering-gallery modes. *Phys. Lett. A*.

[153] Gorodetsky M. L., Savchenkov A. A., Ilchenko V. S. (1996). Ultimate Q of optical microsphere resonators. *Opt. Lett.*.

[154] Armani D. K., Kippenberg T. J., Spillane S. M., Vahala K. J. (2003). Ultra-high-Q toroid microcavity on a chip. *Nature*.

[155] Pasquazi A. (2018). Micro-combs: a novel generation of optical sources. *Phys. Rep.*.

[156] Almeida V. R., Barrios C. A., Panepucci R. R., Lipson M. (2004). All-optical control of light on a silicon chip. *Nature*.

[157] Kippenberg T., Spillane S., Vahala K. (2004). Kerr-nonlinearity optical parametric oscillation in an ultrahigh-Q toroid microcavity. *Phys. Rev. Lett.*.

[158] Savchenkov A. A., Matsko A. B., Strekalov D., Mohageg M., Ilchenko V. S., Maleki L. (2004). Low threshold optical oscillations in a whispering gallery mode CaF_2_ resonator. *Phys. Rev. Lett.*.

[159] Del’Haye P., Schliesser A., Arcizet O., Wilken T., Holzwarth R., Kippenberg T. J. (2007). Optical frequency comb generation from a monolithic microresonator. *Nature*.

[160] Ferrera M. (2008). Low-power continuous-wave nonlinear optics in doped silica glass integrated waveguide structures. *Nat. Photonics*.

[161] Turner A. C., Foster M. A., Gaeta A. L., Lipson M. (2008). Ultra-low power parametric frequency conversion in a silicon microring resonator. *Opt. Express*.

[162] Razzari L. (2010). CMOS-compatible integrated optical hyper-parametric oscillator. *Nat. Photonics*.

[163] Levy J. S., Gondarenko A., Foster M. A., Turner-Foster A. C., Gaeta A. L., Lipson M. (2010). CMOS-compatible multiple-wavelength oscillator for on-chip optical interconnects. *Nat. Photonics*.

[164] Del’Haye P., Herr T., Gavartin E., Gorodetsky M. L., Holzwarth R., Kippenberg T. J. (2011). Octave spanning tunable frequency comb from a microresonator. *Phys. Rev. Lett.*.

[165] Okawachi Y., Saha K., Levy J. S., Wen Y. H., Lipson M., Gaeta A. L. (2011). Octave-spanning frequency comb generation in a silicon nitride chip. *Opt. Lett.*.

[166] Ferdous F. (2011). Spectral line-by-line pulse shaping of on-chip microresonator frequency combs. *Nat. Photonics*.

[167] Johnson A. R. (2012). Chip-based frequency combs with sub-100 GHz repetition rates. *Opt. Lett.*.

[168] Peccianti M. (2012). Demonstration of a stable ultrafast laser based on a nonlinear microcavity. *Nat. Commun.*.

[169] Pasquazi A., Peccianti M., Little B. E., Chu S. T., Moss D. J., Morandotti R. (2012). Stable, dual mode, high repetition rate mode-locked laser based on a microring resonator. *Opt. Express*.

[170] Herr T. (2012). Universal formation dynamics and noise of Kerr-frequency combs in microresonators. *Nat. Photonics*.

[171] Saha K. (2013). Modelocking and femtosecond pulse generation in chip-based frequency combs. *Opt. Express*.

[172] Herr T. (2014). Temporal solitons in optical microresonators. *Nat. Photonics*.

[173] Xue X. (2015). Mode-locked dark pulse Kerr combs in normal-dispersion microresonators. *Nat. Photonics*.

[174] Brasch V. (2016). Photonic chip-based optical frequency comb using soliton Cherenkov radiation. *Science*.

[175] Del’Haye P. (2016). Phase-coherent microwave-to-optical link with a self-referenced microcomb. *Nat. Photonics*.

[176] Bao C. (2017). Dual-pump generation of high-coherence primary Kerr combs with multiple sub-lines. *Opt. Lett.*.

[177] Li Q. (2017). Stably accessing octave-spanning microresonator frequency combs in the soliton regime. *Optica*.

[178] Stern B., Ji X., Okawachi Y., Gaeta A. L., Lipson M. (2018). Battery-operated integrated frequency comb generator. *Nature*.

[179] Jin W. (2021). Hertz-linewidth semiconductor lasers using CMOS-ready ultra-high-Q microresonators. *Nat. Photonics*.

[180] Anderson M. H. (2021). Photonic chip-based resonant supercontinuum via pulse-driven Kerr microresonator solitons. *Optica*.

[181] Xiang C. (2021). Laser soliton microcombs heterogeneously integrated on silicon. *Science*.

[182] Lihachev G. (2022). Platicon microcomb generation using laser self-injection locking. *Nat. Commun.*.

[183] Xie Y. (2022). Soliton frequency comb generation in CMOS-compatible silicon nitride microresonators. *Photonics Res.*.

[184] Helgason Ó. B., Girardi M., Ye Z., Lei F., Schröder J., Torres-Company V. (2023). Surpassing the nonlinear conversion efficiency of soliton microcombs. *Nat. Photonics*.

[185] Li J. (2023). Symmetrically dispersion-engineered microcombs. *Commun. Phys.*.

[186] Miao R. (2024). Dual-microcomb generation via a monochromatically pumped dual-mode microresonator. *Photonics Res.*.

[187] Sun Z., Li Y., Bai B., Zhu Z., Sun H.-B. (2022). Silicon nitride-based Kerr frequency combs and applications in metrology. *Adv. Photonics*.

[188] Yi X., Yang Q.-F., Yang K. Y., Suh M.-G., Vahala K. (2015). Soliton frequency comb at microwave rates in a high-Q silica microresonator. *Optica*.

[189] Griffith A. G. (2015). Silicon-chip mid-infrared frequency comb generation. *Nat. Commun.*.

[190] Guidry M. A. (2020). Optical parametric oscillation in silicon carbide nanophotonics. *Optica*.

[191] Wang C. (2021). High-Q microresonators on 4H-silicon-carbide-on-insulator platform for nonlinear photonics. *Light: Sci. Appl.*.

[192] Chang L. (2020). Ultra-efficient frequency comb generation in AlGaAs-on-insulator microresonators. *Nat. Commun.*.

[193] Gong Z., Liu X., Xu Y., Tang H. X. (2020). Near-octave lithium niobate soliton microcomb. *Optica*.

[194] Hausmann B. J. M., Bulu I., Venkataraman V., Deotare P., Lončar M. (2014). Diamond nonlinear photonics. *Nat. Photonics*.

[195] Yu S.-P., Lucas E., Zang J., Papp S. B. (2022). A continuum of bright and dark-pulse states in a photonic-crystal resonator. *Nat. Commun.*.

[196] Wilson D. J. (2020). Integrated gallium phosphide nonlinear photonics. *Nat. Photonics*.

[197] Liu X., Gong Z., Bruch A. W., Surya J. B., Lu J., Tang H. X. (2021). Aluminum nitride nanophotonics for beyond-octave soliton microcomb generation and self-referencing. *Nat. Commun.*.

[198] Webb K. E., Erkintalo M., Coen S., Murdoch S. G. (2016). Experimental observation of coherent cavity soliton frequency combs in silica microspheres. *Opt. Lett.*.

[199] Lee S. H. (2017). Towards visible soliton microcomb generation. *Nat. Commun.*.

[200] Ma J. (2019). Visible Kerr comb generation in a high-Q silica microdisk resonator with a large wedge angle. *Photonics Res.*.

[201] Chen D., Kovach A., Shen X., Poust S., Armani A. M. (2017). On-chip ultra-high-Q silicon oxynitride optical resonators. *ACS Photonics*.

[202] Chen D., Kovach A., Poust S., Gambin V., Armani A. M. (2019). Normal dispersion silicon oxynitride microresonator Kerr frequency combs. *Appl. Phys. Lett.*.

[203] Kovach A. (2020). Emerging material systems for integrated optical Kerr frequency combs. *Adv. Opt. Photonics*.

[204] Liu J. (2022). Emerging material platforms for integrated microcavity photonics. *Sci. China: Phys., Mech. Astron.*.

[205] Zhou H. (2019). Soliton bursts and deterministic dissipative Kerr soliton generation in auxiliary-assisted microcavities. *Light: Sci. Appl.*.

[206] Wan S. (2020). Frequency stabilization and tuning of breathing solitons in Si_3_N_4 _microresonators. *Photonics Res.*.

[207] Marin-Palomo P. (2017). Microresonator-based solitons for massively parallel coherent optical communications. *Nature*.

[208] Cheng J. (2023). Human emotion recognition with a microcomb-enabled integrated optical neural network. *Nanophotonics*.

[209] Yu M., Okawachi Y., Griffith A. G., Lipson M., Gaeta A. L. (2016). Mode-locked mid-infrared frequency combs in a silicon microresonator. *Optica*.

[210] Cai L., Li J., Wang R., Li Q. (2022). Octave-spanning microcomb generation in 4H-silicon-carbide-on-insulator photonics platform. *Photonics Res.*.

[211] Wang S. (2013). 4H‐SiC: a new nonlinear material for midinfrared lasers. *Laser Photonics Rev.*.

[212] Pu M., Ottaviano L., Semenova E., Yvind K. (2016). Efficient frequency comb generation in AlGaAs-on-insulator. *Optica*.

[213] Moille G. (2020). Dissipative kerr solitons in a III-V microresonator. *Laser Photonics Rev.*.

[214] Xie W., Xiang C., Chang L., Jin W., Peters J., Bowers J. E. (2022). Silicon-integrated nonlinear III-V photonics. *Photonics Res.*.

[215] He Y. (2019). Self-starting bi-chromatic LiNbO_3_ soliton microcomb. *Optica*.

[216] Yu M. (2020). Raman lasing and soliton mode-locking in lithium niobate microresonators. *Light: Sci. Appl.*.

[217] Xie R.-R., Li G.-Q., Chen F., Long G.-L. (2021). Microresonators in lithium niobate thin films. *Adv. Opt. Mater.*.

[218] Jung H., Yu S.-P., Carlson D. R., Drake T. E., Briles T. C., Papp S. B. (2021). Tantala kerr nonlinear integrated photonics. *Optica*.

[219] Lucas E., Yu S.-P., Briles T. C., Carlson D. R., Papp S. B. (2023). Tailoring microcombs with inverse-designed, meta-dispersion microresonators. *Nat. Photonics*.

[220] Jung H., Xiong C., Fong K. Y., Zhang X., Tang H. X. (2013). Optical frequency comb generation from aluminum nitride microring resonator. *Opt. Lett.*.

[221] Liu K. (2023). Mitigating fast thermal instability by engineered laser sweep in AlN soliton microcomb generation. *Photonics Res.*.

[222] Lamb E. S., Carlson D. R., Hickstein D. D., Stone J. R., Diddams S. A., Papp S. B. (2018). Optical-frequency measurements with a Kerr microcomb and photonic-chip supercontinuum. *Phys. Rev. Appl.*.

[223] Parriaux A., Hammani K., Millot G. (2020). Electro-optic frequency combs. *Adv. Opt. Photonics*.

[224] Ren T. (2019). An integrated low-voltage broadband lithium niobate phase modulator. *IEEE Photonics Technol. Lett.*.

[225] Zhang K. (2023). A power-efficient integrated lithium niobate electro-optic comb generator. *Commun. Phys.*.

[226] Renaud D. (2023). Sub-1 Volt and high-bandwidth visible to near-infrared electro-optic modulators. *Nat. Commun.*.

[227] Zhuang R. (2023). Electro‐optic frequency combs: theory, characteristics, and applications. *Laser Photonics Rev.*.

[228] Zhang M. (2019). Broadband electro-optic frequency comb generation in a lithium niobate microring resonator. *Nature*.

[229] Hu Y. (2022). High-efficiency and broadband on-chip electro-optic frequency comb generators. *Nat. Photonics*.

[230] Shams-Ansari A. (2022). Thin-film lithium-niobate electro-optic platform for spectrally tailored dual-comb spectroscopy. *Commun. Phys.*.

[231] Wei W., Chen J., Huang J., Wang Z., Zhang J., Wang T. (2022). Advances of semiconductor mode-locked laser for optical frequency comb generation. *Natl. Sci. Open*.

[232] Hargrove L., Fork R. L., Pollack M. (1964). Locking of He-Ne laser modes induced by synchronous intracavity modulation. *Appl. Phys. Lett.*.

[233] Ho P. T., Glasser L., Ippen E., Haus H. (1978). Picosecond pulse generation with a cw GaAlAs laser diode. *Appl. Phys. Lett.*.

[234] Ippen E., Eilenberger D., Dixon R. (1980). Picosecond pulse generation by passive mode locking of diode lasers. *Appl. Phys. Lett.*.

[235] Huang X., Stintz A., Li H., Lester L., Cheng J., Malloy K. (2001). Passive mode-locking in 1.3 μm two-section InAs quantum dot lasers. *Appl. Phys. Lett.*.

[236] Koch B. R., Fang A. W., Cohen O., Bowers J. E. (2007). Mode-locked silicon evanescent lasers. *Opt. Express*.

[237] Hugi A., Villares G., Blaser S., Liu H. C., Faist J. (2012). Mid-infrared frequency comb based on a quantum cascade laser. *Nature*.

[238] Villares G. (2015). On-chip dual-comb based on quantum cascade laser frequency combs. *Appl. Phys. Lett.*.

[239] Wang Z. (2017). A III-V-on-Si ultra-dense comb laser. *Light: Sci. Appl.*.

[240] Meng B. (2020). Mid-infrared frequency comb from a ring quantum cascade laser. *Optica*.

[241] Jaidl M. (2021). Comb operation in terahertz quantum cascade ring lasers. *Optica*.

[242] Dong B., Dumont M., Terra O., Wang H., Netherton A., Bowers J. E. (2023). Broadband quantum-dot frequency-modulated comb laser. *Light: Sci. Appl.*.

[243] Kazakov D. (2024). Active mid-infrared ring resonators. *Nat. Commun.*.

[244] Chai Z., Hu X., Wang F., Niu X., Xie J., Gong Q. (2017). Ultrafast all‐optical switching. *Adv. Opt. Mater.*.

[245] Martínez A. (2010). Ultrafast all-optical switching in a silicon-nanocrystal-based silicon slot waveguide at telecom wavelengths. *Nano Lett.*.

[246] Takiguchi M. (2020). All-optical InAsP/InP nanowire switches integrated in a Si photonic crystal. *ACS Photonics*.

[247] Guo Q. (2022). Femtojoule femtosecond all-optical switching in lithium niobate nanophotonics. *Nat. Photonics*.

[248] Hu H., Oxenløwe L. K. (2021). Chip-based optical frequency combs for high-capacity optical communications. *Nanophotonics*.

[249] Sun Y. (2023). Applications of optical microcombs. *Adv. Opt. Photonics*.

[250] Pfeifle J. (2014). Coherent terabit communications with microresonator Kerr frequency combs. *Nat. Photonics*.

[251] Hu H. (2018). Single-source chip-based frequency comb enabling extreme parallel data transmission. *Nat. Photonics*.

[252] Fülöp A. (2018). High-order coherent communications using mode-locked dark-pulse Kerr combs from microresonators. *Nat. Commun.*.

[253] Corcoran B. (2020). Ultra-dense optical data transmission over standard fibre with a single chip source. *Nat. Commun.*.

[254] Geng Y. (2022). Coherent optical communications using coherence-cloned Kerr soliton microcombs. *Nat. Commun.*.

[255] Cheng Q., Bahadori M., Glick M., Rumley S., Bergman K. (2018). Recent advances in optical technologies for data centers: a review. *Optica*.

[256] Miller D. A. J. P. O. T. I. (2000). Rationale and challenges for optical interconnects to electronic chips. *Proc. IEEE*.

[257] Okawachi Y., Kim B. Y., Lipson M., Gaeta A. L. (2023). Chip-scale frequency combs for data communications in computing systems. *Optica*.

[258] Rizzo A. (2023). Massively scalable Kerr comb-driven silicon photonic link. *Nat. Photonics*.

[259] Bai B. (2023). Microcomb-based integrated photonic processing unit. *Nat. Commun.*.

[260] Shen Y., Harris N. C., Skirlo S., SoljaCiC M. (2017). Deep learning with coherent nanophotonic circuits. *Nat. Photonics*.

[261] Lin X. (2018). All-optical machine learning using diffractive deep neural networks. *Science*.

[262] Feldmann J., Youngblood N., Wright C. D., Bhaskaran H., Pernice W. H. P. (2019). All-optical spiking neurosynaptic networks with self-learning capabilities. *Nature*.

[263] Ashtiani F., Geers A. J., Aflatouni F. J. N. (2022). An on-chip photonic deep neural network for image classification. *Nature*.

[264] Zhang J. (2023). Dual optical frequency comb neuron: Co-developing hardware and algorithm. *Adv. Intell. Syst.*.

[265] Yin R. (2023). Integrated WDM-compatible optical mode division multiplexing neural network accelerator. *Optica*.

[266] Li J., Yi X., Lee H., Diddams S. A., Vahala K. J. (2014). Electro-optical frequency division and stable microwave synthesis. *Science*.

[267] Tetsumoto T., Nagatsuma T., Fermann M. E., Navickaite G., Geiselmann M., Rolland A. (2021). Optically referenced 300 GHz millimetre-wave oscillator. *Nat. Photonics*.

[268] Sun S. (2024). Integrated optical frequency division for microwave and mmWave generation. *Nature*.

[269] Kudelin I. (2024). Photonic chip-based low-noise microwave oscillator. *Nature*.

[270] Zhao Y. (2024). All-optical frequency division on-chip using a single laser. *Nature*.

[271] Wang J., Sciarrino F., Laing A., Thompson M. G. (2020). Integrated photonic quantum technologies. *Nat. Photonics*.

[272] Silverstone J. W. (2014). On-chip quantum interference between silicon photon-pair sources. *Nat. Photonics*.

[273] Reimer C. (2016). Generation of multiphoton entangled quantum states by means of integrated frequency combs. *Science*.

[274] Kues M. (2017). On-chip generation of high-dimensional entangled quantum states and their coherent control. *Nature*.

[275] Autebert C. (2016). Integrated AlGaAs source of highly indistinguishable and energy-time entangled photons. *Optica*.

[276] Zhao J., Ma C., Rüsing M., Mookherjea S. (2020). High quality entangled photon pair generation in periodically poled thin-film lithium niobate waveguides. *Phys. Rev. Lett.*.

[277] Wang J. (2018). Multidimensional quantum entanglement with large-scale integrated optics. *Science*.

[278] Elshaari A. W., Pernice W., Srinivasan K., Benson O., Zwiller V. (2020). Hybrid integrated quantum photonic circuits. *Nat. Photonics*.

[279] Zhou Z. (2023). Prospects and applications of on-chip lasers. *Elight*.

[280] Moody G., Chang L., Steiner T. J., Bowers J. E. (2020). Chip-scale nonlinear photonics for quantum light generation. *AVS Quantum Sci.*.

[281] Eggleton B. J. (2012). Photonic chip based ultrafast optical processing based on high nonlinearity dispersion engineered chalcogenide waveguides. *Laser Photonics Rev.*.

